# The Underexplored Landscape of Hypoxia-Inducible Factor 2 Alpha and Potential Roles in Tumor Macrophages: A Review

**DOI:** 10.3390/oxygen3010005

**Published:** 2023-01-31

**Authors:** Kayla J. Steinberger, Timothy D. Eubank

**Affiliations:** 1 Department of Microbiology, Immunology, and Cell Biology, West Virginia University, Morgantown, WV 26505, USA; 2 In Vivo Multifunctional Magnetic Resonance Center, West Virginia University, Morgantown, WV 26505, USA; 3 West Virginia University Cancer Institute, Morgantown, WV 26505, USA

**Keywords:** hypoxia, macrophage, tumor, HIF, cancer, angiogenesis, myeloid cell, oxygen

## Abstract

Low tissue oxygenation, termed *hypoxia*, is a characteristic of solid tumors with negative consequences. Tumor-associated macrophages (TAMs) accumulate in hypoxic tumor regions and correlate with worse outcomes in cancer patients across several tumor types. Thus, the molecular mechanism in which macrophages respond to low oxygen tension has been increasingly investigated in the last decade. Hypoxia stabilizes a group of hypoxia-inducible transcription factors (HIFs) reported to drive transcriptional programs involved in cell survival, metabolism, and angiogenesis. Though both tumor macrophage HIF-1α and HIF-2α correlate with unfavorable tumor microenvironments, most research focuses on HIF-1α as the master regulator of hypoxia signaling, because HIF-1α expression was originally identified in several cancer types and correlates with worse outcome in cancer patients. The relative contribution of each HIFα subunit to cell phenotypes is poorly understood especially in TAMs. Once thought to have overlapping roles, recent investigation of macrophage HIF-2α has demonstrated a diverse function from HIF-1α. Little work has been published on the differential role of hypoxia-dependent macrophage HIF-2α when compared to HIF-1α in the context of tumor biology. This review highlights cellular HIF-2α functions and emphasizes the gap in research investigating oxygen-dependent functions of tumor macrophage HIF-2α.

## Graphical Abstract

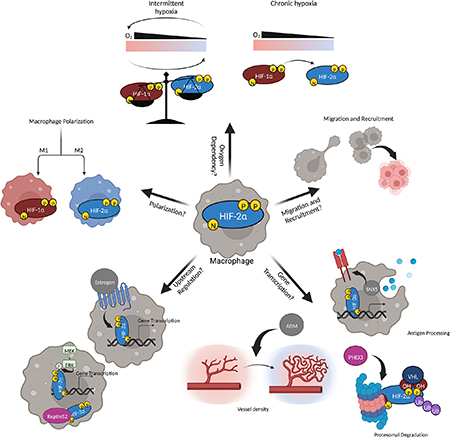

## 1. Introduction

Low tissue oxygenation, termed *hypoxia*, is a notorious characteristic of solid tumors [1]. As a response to increased cellular proliferation rates or tumor growth in regions devoid of blood vessels, tumors attempt to recover oxygen by sending signals for increased blood vessel formation via angiogenesis—the growth of new vessels from pre-existing vasculature. While newly formed blood vessels during development form an ideally spaced, structured vessel tree efficient in oxygen delivery and blood perfusion, newly formed tumor blood vessels are disorganized and lack structural integrity in human tumors due to the heterogeneous overexpression of vascular endothelial growth factor (VEGF) resulting in inadequate vessel perfusion and transient hypoxia [2]. Tumor-associated macrophages (TAMs) accumulate in hypoxic tumor regions and regulate dysfunctional angiogenesis by secreting pro-angiogenic factors such as VEGF [3]. Thus, the molecular mechanism in which macrophages respond to low oxygen tension has been increasingly investigated in the last decade.

**Figure 1.**
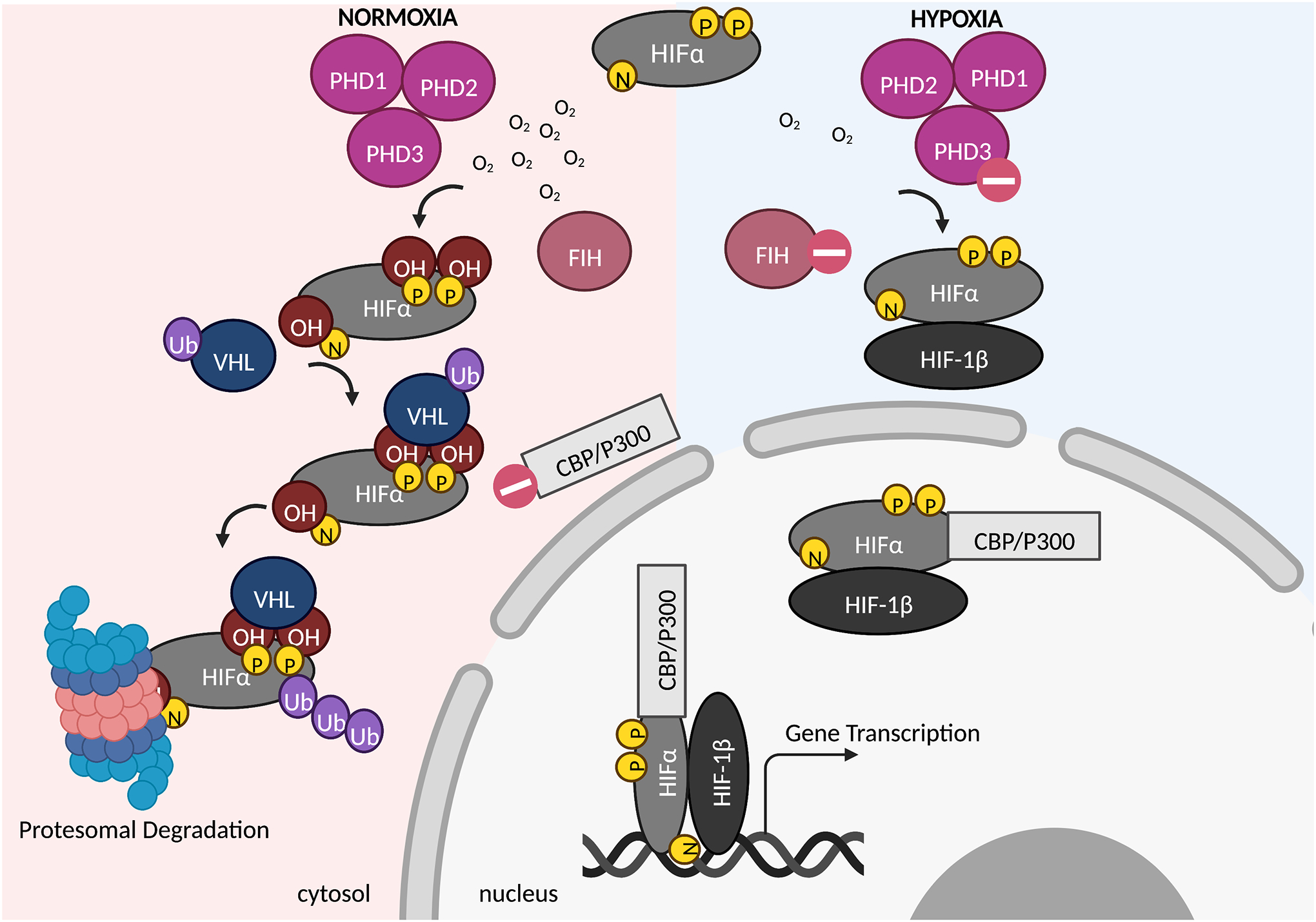
HIF degradation and stabilization. During normoxia, when oxygen is readily available, HIFα subunits are hydroxylated at prolyl residues (P) by prolyl hydroxylases (PHD1, −2, and 3) or at an asparagine residue (N) by factor inhibiting HIF (FIH). Hydroxylation prevents binding of 300-kilodalton coactivator protein (p300) and CREB binding protein (CBP). E3 ubiquitin ligase von Hippel-Lindau (VHL) polyubiquitinates hydroxylated HIFα for proteasomal degradation. Hypoxia prevents HIFα hydroxylation resulting in stabilization in the cytoplasm. HIFα subunits heterodimerize with HIF-1β and translocate to the cell nucleus where CBPp300 binds HIFα. This complex enhances transcription.

Hypoxia stabilizes a group of hypoxia-inducible transcription factors (HIFs) reported to drive transcriptional programs involved in cell survival [4], metabolism [5–7], and angiogenesis [8–11]. HIF-1α and HIF-2α are basic helix-loop-helix (bHLH)-PAS domain transcription factors which are heavily regulated at the protein level (Figure 1). During physioxic conditions (normal oxygen levels for a specific tissue), HIFα subunits are hydroxylated at specific proline residues by prolyl hydroxylases (PHD1, −2, and 3) or at an asparagine residue by factor inhibiting HIF (FIH) and subsequently targeted by the E3 ubiquitin ligase von Hippel-Lindau (VHL) for polyubiquitination and proteasomal degradation [12–14]. For HIF-1α, this occurs in minutes [15]. PHD enzymes have specificity for the HIFα isoforms with PHD2 having more influence on HIF-1α than HIF-2α and PHD3 having more influence on HIF-2α than HIF-1α [16]. HIF-1α is hydroxylated at proline residues 402, 564, or both preferentially by PHD2, or at asparagine residue 803 by FIH [17]. HIF-2α is hydroxylated at proline residue 405, 531, or both preferentially by PHD3 or at asparagine residue 851 by FIH. Hydroxylation prevents the binding of 300-kilodalton coactivator protein (p300) and CREB binding protein (CBP). HIFα is then ubiquitinated by VHL and degraded. Low oxygenation prevents HIFα hydroxylation and thus stabilizes these transcription factors. They accumulate in the cytoplasm then translocate to the nucleus, binding to CBP/p300. Acting as heterodimers, hypoxia-stabilized HIF-1α or HIF-2α/EPAS1 (endothelial PAS domain protein 1) bind to constitutive aryl hydrocarbon receptor nuclear translocator (ARNT)/HIF-1β and accumulate in the cell nucleus to stimulate transcription at conserved DNA sequences, hypoxia response elements (HREs, consensus pattern (5′-RCGTG-3′) located near promoters or enhancers and associate with hypoxia accessory sequences (HAS, consensus pattern (5′-CA(G|C)(A|G)(T|G|C)-3′) immediately downstream (within 15 nucleotides) [18–20].

In addition to HIF-1α and HIF-2α, there is also a lesser studied HIF-3α that has high homology ((bHLH)-PAS) to HIF-1α and HIF-2α sans C-terminal transactivation domain (C-TAD) and similarly ubiquitinated in an oxygen-dependent manner by VHL [21,22]. The biological function of HIF-3 is underexplored, and its expression is seemingly tissue restricted [23–25]. HIF-3α has been shown to act as a suppressor of hypoxic gene induction, working in opposition to HIF-1α and HIF-2α [21,24,26,27]. For example, inhibition of HIF-3α with siRNA increases stem progenitor cell recruitment while siRNA to HIF-1α and HIF-2α reduces recruitment [28]. More recent work suggests HIF-3α can act as an oxygen-dependent transcription factor and increase gene expression [29,30].

The relative contribution of each HIFα subunit to hypoxic-driven cell phenotypes is poorly understood especially in tumor macrophages. Most research focuses on HIF-1α as the master regulator of hypoxia signaling [31]. HIF-1α expression was originally identified in several cancer types and in metastases, suggesting a role for HIFs in tumor progression [32] and worse outcome in patients [33–39]. Once thought to have overlapping roles, more recent investigation of macrophage HIF-2α has demonstrated a diverse function from HIF-1α. No studies published have investigated macrophage HIF-3α, thus this review will focus on HIF-1α and HIF-2α. Little work has been published on the differential role of hypoxia-dependent macrophage HIF-2α when compared to HIF-1α in the context of tumor biology. The objective of this review is to highlight myeloid HIF-2α functions and emphasize the gap in research investigating oxygen-dependent functions regulated by tumor macrophage HIF-2α.

## 2. HIF-2α in Different Cell Types

### 2.1. Endothelium

**Figure 2.**
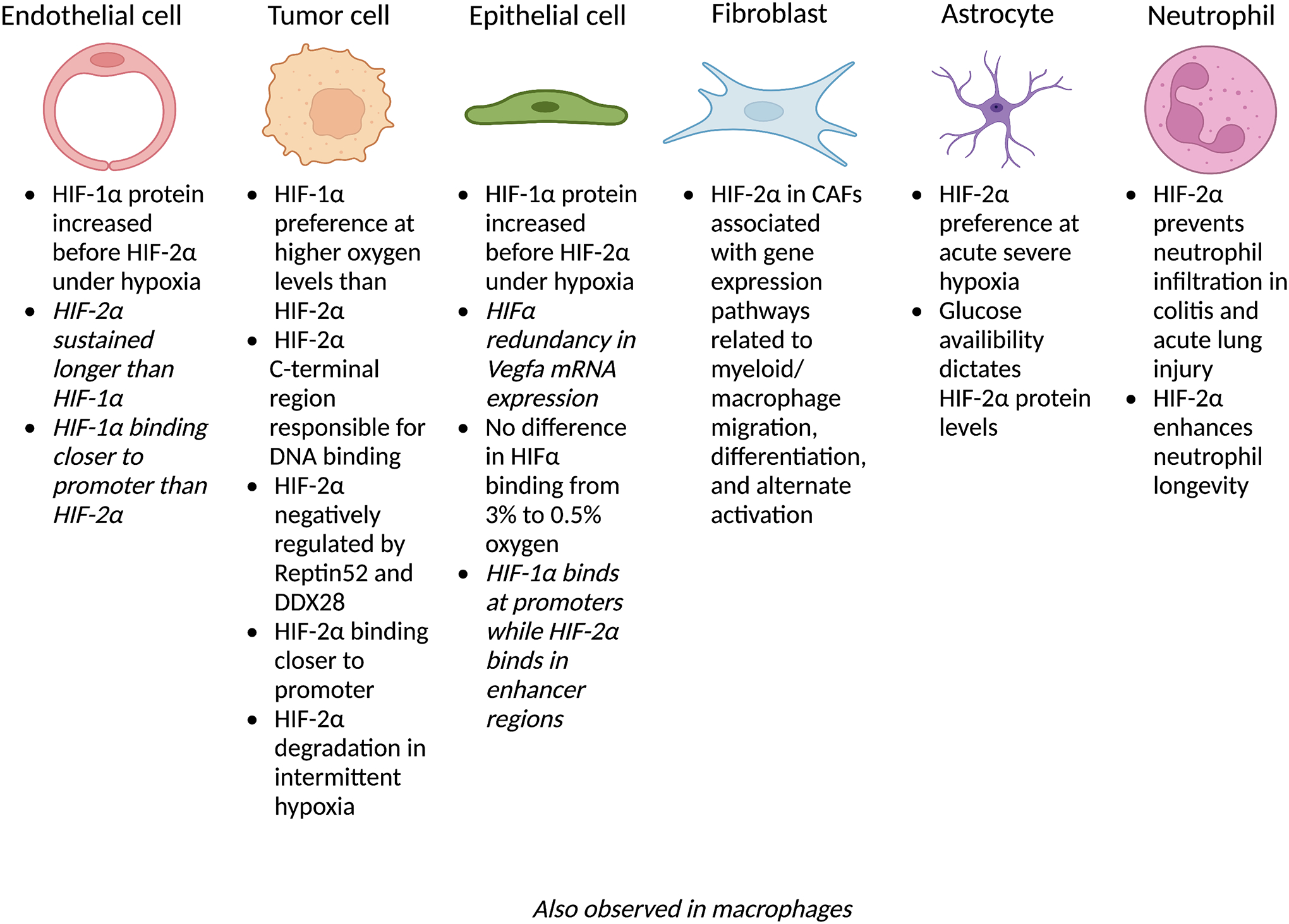
HIFα in different cell types. Known characteristics of HIF-2α in non-macrophage cell types and shared characteristics observed in macrophages (*italicized*). DEAD Box protein 28 (DDX28). Cancer-associated fibroblast (CAF).

Preferential HIF-mediated transcription may be dictated by the time and intensity of hypoxia experienced by cells within the tumor microenvironment. In one study investigating HIF-1α versus HIF-2α stabilization in ten human primary endothelial cell (EC) types, HIF-1α rapidly accumulated in all tested ECs reaching maximal levels between 2 to 6 h of hypoxia (0.9% oxygen) and declined by 48 h [40]. HIF-2α gradually increased in all ECs tested and reached maximal levels between 8 and 20 h of hypoxia, and HIF-2α protein expression was sustained over 48 h [40,41], suggesting that HIF-1α may drive initial hypoxia responses (Figure 2).

The transcriptome of a 10-donor pool of human umbilical vein endothelial cells (HUVECs) was analyzed for responses to 2 h, 8 h and 16 h of hypoxia [40]. The analysis revealed a rapid increase in genes affected from 7 to 72 to 280 genes over 2, 8, and 16 h, respectively. The promoters of gene transcripts affected by hypoxia were analyzed for HIF-1α or HIF-2α binding sites (HRE motifs) in open chromatin regions of a 20-kb window around the transcription start site (ENCODE project). HRE motifs were originally identified with a consensus core sequence (5′-RCGTG-3′) and are readily observed within 1 kb upstream from the transcriptional start site [42]. Using chromatin immunoprecipitation sequencing (ChIP-seq) results from Smythies et al. [43], each HIFα was found to load progressively at a distinct repertoire of sites across the genome with marked biases of HIF-1α binding proximal to the transcription start site and HIF-2α binding distal to the transcription start site (~90% sites > 5 kb beyond the core HRE sequence) [43]. For each gene identified, the counts of HREs found in the open chromatin regions were reported as a cumulative distribution function on the three time-point group of genes. As expected, 230 of the 232 genes affected during hypoxia contained HRE motifs in their promoter regions and in general, genes that were affected earlier had more HRE binding regions than those affected later [27]. The HRE genes affected by acute hypoxia (2 h) had promoter regions enriched with HIF-1α motifs which resemble the expected HRE core sequence [HIF-1α motif core sequence: 5′-(A/G)CGTG-3′] while genes affected during prolonged hypoxia (8 h) had more HIF-2α motifs which, though similar, still vary considerably from the established HRE core sequence [HIF-2α motif core sequence: 5′-(G/A)(T/C)(A/G)(C/G)G(T/A)] in their promoter regions. This suggests that the hypoxic-driven transcriptional profiling changes over time are coordinated by a HIFα switch and further supports HIF-1α preference during early hypoxia [27,31–33]. Upon HIF-2α silencing in human ECs, there was a small increase in *HIF1A* mRNA levels (~ 1.3-fold) and an increase (though insignificant) in HIF-1α protein, suggesting that HIF-2α may suppress HIF-1α [28]. Similarly, silencing *HIF1A* (HIF-1α) led to a small reduction of *EPAS1* mRNA, but this was not reflected at all in HIF-2α protein levels, suggesting that HIF-1α and HIF-2α subunits may limit mRNA transcription of the other but only HIF-2α may have an effect at the protein level.

Given that HIF-1α protein declines faster than HIF-2α in human ECs under hypoxia, the stability of *HIF1A* and *EPAS1* mRNA may be different. The *EPAS1* 3′UTR is less prone to Adenylate-Uridylate-Rich Element (ARE)-dependent destabilization than *HIF1A* mRNA [40]. However, this only represents one destabilizing mechanism and could be explained by other stabilizing and destabilizing processes and warrants further investigation. This stability divergence is also recapitulated in human macrophages. HIF-1α protein is increased in monocyte-derived macrophages (MDMs) starting at 1 h of hypoxia and declines by 24 h [44]. On the other hand, HIF-2α is rapidly increased by 1 h of hypoxia and only slightly declines at 24 h, suggesting a possible preferential switch from HIF-1α to HIF-2α during chronic episodes of hypoxia. While HIF-1α expression rapidly degraded with re-oxygenation, HIF-2α remained elevated 2 h after re-oxygenation suggesting that HIF-2α may be more stable or more resistant to mRNA/protein degradation than HIF-1α [44].

In human ECs, several genes are transcribed by HIF-1α or HIF-2α preferentially. For example, the induction of *Ankyrin Repeat Domain 37* (*ANKRD37*), *Bcl2-interacting Protein 3* (*BNIP3*), *Nuclear Prelamin A Recognition Factor* (*NARF*), and *Solute Carrier Family 2 Member 1* (*SLC2A1*) were dependent on HIF-1α while *Adrenomedullin* (*ADM*), *Angiopoietin-like 4* (*ANGPTL4*), *Chromosome 1 open reading frame 21* (*C1orf21*), *membrane-associated guanylate kinase* (*MAGUK*), and *PDX Domain-containing 1* (MAGI1), and *Prostaglandin I2 Synthase* (*PTGIS*) were regulated by HIF-2α. *BCL2 Interacting Protein Like* (*BNIPL*), *Egl-9 Family Hypoxia-inducible Factor 3* (*EGLN3* or *PHD3*), *Lung Cancer-Associated Transcript 1* (*LUCAT1*), and *MIR210 Host Gene* (*MIR210HG*) were decreased when either HIF-1α or −2α were knocked down suggesting redundancy. *EGLN3* is seemingly regulated by both HIF-1α and −2α in HUVECs and mouse macrophages [45–47]. Knockdown of HIF-2α increases induction of HIF-1α-regulated genes *NARF* (at 2 h hypoxia) and *BNIP3* (at 8 h hypoxia), suggesting a possible effect of HIF-2α suppression of HIF-1α function as HIF-1α protein is slightly increased by HIF-2α knockdown [48]. Table 1 indicates genes preferentially driven by HIF-2α in different cell types which may help delineate cell-specific HIFα activity.

Limited work has been done on endothelial HIF-2α, in vivo. In terms of gene expression, ex vivo isolated primary pulmonary endothelial cells have reduced *Arg1* mRNA expression in mice with endothelial HIF-2α deficiency, but it is unclear if this is affected by HIF-1α [65]. Arginase 1 is reported to be an anti-inflammatory marker as it competes with pro-inflammatory inducible nitric oxide synthase (iNOS) for L-arginine metabolism. Interestingly, hypoxic (1.5% for 16 h) induction of pro-inflammatory *genes stromal cell-derived factor 1 (SDF1)*, C-X-C motif chemokine receptor 4 *(CXCR4)*, intercellular adhesion molecule1 *(ICAM1)* and transforming growth factor alpha *(TGFA)* in normal human pulmonary-derived ECs was also prevented by the small molecule HIF-2α inhibitor PT2567 and HIF-2α siRNA but not HIF-1α siRNA [52], suggesting HIF-2α-mediated expression of these genes. However, their functional relevance remains unexplored.

### 2.2. Tumor Cells

Inactivation of the *VHL* tumor suppressor gene is the signature initiating event in clear cell renal cell carcinoma (ccRCC), which is the most common form of kidney cancer [66]. *VHL* mutation leads to abnormal constitutive stabilization of HIF-1α and HIF-2α proteins in normal oxygen conditions. Thus, HIF-2α has been studied primarily in transformed cells in the context of promoting cell survival and proliferation especially in RCC. The phenotypes found in cells with constitutive HIF-2α may provide insight into its function in TAMs.

Not all RCC cell lines are VHL-deficient and, even of those that are, are not ubiquitously dependent on HIF-2α. For example, in the RCC cell line Caki-1 which is VHL-proficient, relative HIF-1α and HIF-2α protein levels increased with subsequent decreased oxygen conditions starting at 10% oxygen for HIF-1α and 1% for HIF-2α with highest detection for both HIF-α subunits at 0.1% oxygen, suggesting HIF-1α preference at higher oxygen concentrations [67]. In contrast, the VHL-defective RCC cell lines Caki-2 and 786–0 have extremely divergent HIFα expression [67]. The Caki-2 RCC cell line had similar levels of sustained HIF-1α protein expression across oxygen concentrations ranging from 20% down to 0.1% oxygen while HIF-2α was undetected at all oxygen levels. In almost complete opposition, 786–0 cells had undetectable HIF-1α and similar levels of sustained HIF-2α protein expression across oxygen concentrations [67]. Thus, certain RCC cell lines are not appropriate for studying HIF-2α. Upstream signaling experimentation in VHL-mutant RCC 786-O cells which have sustained HIF-2α protein expression revealed that estrogen receptor (Erβ) upregulates HIF-2α mRNA and protein through hypothesized transcriptional regulation at the HIF-2α promoter, suggesting partial dependence on the ER-pathway [68].

RCC has also been used to investigate the contribution of HIF-1α and HIF-2α DNA binding/heterodimerization and transactivation domains for target specificity [66]. HIF-1α and HIF-2α have a high degree of amino acid similarity in their N-terminal half containing the basic helix-loop-helix (bHLH) domain involved in DNA binding and the Per-Arnt-Sim (PAS) domain for HIF-1β heterodimerization [69,70]. Less similarity is found in the C-terminal region containing the N-terminal transactivation domain (NTAD) and C-terminal transactivation domain (CTAD) [69]. For a schematic representation of these domains, the motivated reader is directed to Hu et al. [69]. In human RCC WT8 cells, the NTAD/CTAD transactivation region of HIF-2α is more relevant for HIF-2α target selectivity than bHLH/PAS while some HIF-1α-driven gene expression required both NTAD/CTAD and bHLH/PAS. Gene specificity, especially for HIF-2α selective target *PHD3*, could be completely attributed to the C-terminal region of HIF-2α in WT8 RCC cells [66]. This suggests that HIFα target specificity is likely dependent on the C-terminal region of HIFα. Identification of HIF-2α-mediated *Phd3* in vivo by this group also suggests a negative feedback mechanism as PHD3 preferentially targets HIF-2α and not HIF-1α for degradation [66,71].

Signaling pathways involving HIF-2α have also been investigated in other tumor cell types. In clear cell carcinoma and colorectal carcinoma, HIF-2α has been shown to play a role cellular iron homeostasis and ferroptosis susceptibility [72,73]. For example, in colorectal cancers, HIF-2α activation potentiates oxidative cell death by increasing cellular iron [72]. Additionally, HeLa cells transfected with Flag-tagged HIF-2α revealed interaction with Reptin52, an ATP-binding protein [74]. Hypoxia resulted in translocation of Reptin52 from nucleus to cytoplasm and increased HIF-2α and Reptin52 colocalization in the cytoplasm upon extracellular signal-regulated kinase (ERK) 1/2 inhibition. This suggests that Reptin52 may reduce HIF-2α nuclear activity by a non-canonical PHD-VHL-proteasome independent mechanism [74].

Another possible negative regulator of HIF-2α was identified in a human glioblastoma cell line. DEAD box protein DDX28 was found to be a negative regulator of HIF-2α and not HIF-1α [75]. The authors proposed that DDX28 sequesters HIF-2α and suppresses its ability to activate eIF4E2 cap binding and translation of eIF4E2 target mRNAs such as the *EPAS1* (HIF-2α) gene itself [75]. This method further complicates how HIF transcription is self-regulated as this study suggests that without DDX28, HIF-2α would drive its own expression (EPAS1) through eIF4E2 in an indirect positive feedback mechanism.

HIF-2α has been associated with both oncogenic and tumor suppressive phenotypes in breast tumor cells. Meta-analysis of primary breast tumors demonstrated that HIF-2α expression was higher in HER2-overexpressing samples when compared to Luminal A, Luminal B and basal subtypes, and survival analysis revealed HIF-2α expression was associated with worse prognosis in patients in the HER2-positive group when compared to HER2-negative [76]. HIF-2α was also higher in HER2-high breast cancer cell lines, suggesting that this effect may be reflective of tumor cell-specific expression of HIF-2α [76]. To understand HIFα targeted genes in human breast cancer cells MDA-MB-231, ChIP was used to find human *CSF1* and *CCR5* genes were bound by both hypoxia-induced HIF-1α and HIF-2α albeit in different locations. For *CSF1*, both hypoxia-induced HIF-1α and HIF-2α bound close to the transcription start site (~600 nucleotides upstream) but only HIF-1α binding occurred farther upstream (~2500 nucleotides upstream). For *CCR5*, only HIF-2α bound close to the transcription start site (~1370 nucleotides upstream) but only HIF-1α bound farther downstream (~8065 nucleotides downstream), suggesting that HIF targeting may depend on DNA-binding location [77]. Interestingly, this opposes results found in human monocyte-derived macrophages (MDMs) which found HIF-1α bound preferentially in promoters while HIF-2α binding was more pronounced in enhancer regions, suggesting that breast tumor cells may not adequately reflect HIFα binding preferences in macrophages [47]. In the breast cancer cell lines MCF-7 and T-57D, shRNA targeting HIF-2α suggested that HIF-2α drives hypoxia-induced WNT1 inducible signaling pathway protein 2 (*WISP2*) expression as it was significantly reduced when HIF-2α was depleted [53], but this effect was not observed in other breast tumor cell lines such as BT-474 and ZR-75–1, suggesting a lack of functional conservation even in similar cancer cell types.

Limited work has investigated HIF-2α-driven expression of non-protein targets. Hypoxia-induced long non-coding RNA (lncRNA) *RAB11B-AS1* is upregulated in numerous human breast cancer cell lines and its expression is induced by canonical HIF-2α signaling but not HIF1α under hypoxic conditions [57]. In vitro, knockdown of lncRNA *RAB11B-AS1* limited cancer cell migration and invasion independent of oxygen while in vivo knockdown decreased microvessel density and metastatic regions in immunodeficient mice, suggesting that HIF-2α may drive an invasive, pro-angiogenic phenotype in breast cancer cells [57]. This contrasts with myeloid specific HIF-2α deficiency which exacerbated tumor growth and increased microvessel density in our murine breast tumor model[9]. Based on our own work in immunocompetent orthotopic PyMT breast tumor-bearing mice, we wonder if these seemingly oncogenic HIF-2α driven effects in tumor cells can be overcome by the tumor suppressive effects seen in myeloid HIF-2α [9].

To add to the complexity of HIFα signaling, HIF-2α may be protective in lung cancer cells. A compensatory effect of HIF-2α when HIF-1α is not present has been shown to protect a non-small cell lung cancer (NSCLC) cell line from radiation under 0.2% oxygen [78]. When HIF-1α was knocked out in H1299 NSCLC cell line, HIF-2α was strongly induced by hypoxia compared to wild-type but the reverse was not seen in HIF-2α knockout (KO) cells. This result, as well as HIF-1α protein expression being significantly higher than HIF-2α in cells with both HIFα’s, suggests HIF-1α may have a suppressive effect on HIF-2α in lung tumor cells with both HIFα’s present. Upstream signaling investigation in human hepatoma Huh7 cells treated with a MEK inhibitor resulted in downregulation of known HIF-2α target genes EPO and PAI-1 (SERPINE1) and shifted hypoxia-stabilized nuclear-localized HIF-2α protein to the cytoplasm. Furthermore, full length HIF-2α was directly phosphorylated by ERK2 in vitro, suggesting that ERK1/2 may stimulate transcriptional activity of HIF-2α during hypoxic conditions [79].

Changing oxygen concentrations which better reflects intermittent perfusion in the tumor microenvironment may provide better insight into the functional role of HIF-2α in TAMs. In rat pheochromocytoma cell lines, HIF-1α is upregulated by intermittent hypoxia (1.5%/20% cycles) while HIF-2α is downregulated. HIF-2α reduction is sequential with increased number of intermittent hypoxia cycles and could recover after 16 h of re-oxygenation. HIF-2α downregulation in these cell lines was a result of calpain protease activation [80]. It was suggested that calpains may selectively target HIF-2α rather than HIF-1α because basal HIF-2α is high in pheochromocytoma PC12 cells, so calpain activation may deplete preexisting HIF-2α protein before de novo HIF-1α protein accumulation occurs resulting in dominate HIF-1α expression. No study to date has investigated intermittent hypoxia on HIF expression in macrophages and further investigation is needed to test this phenomenon in other cell types.

In more sophisticated in vitro models, radiation challenge of small intestinal organoids suggests a protective effect for HIF-2α. Proliferation suppression by prolyl hydroxylase inhibitor FG-4592 on the small intestinal organoids was inhibited by the HIF-2α inhibitor PT2385. Further investigation of the activation of Wnt/β-catenin pathway also verified that PT2385 could significantly block the up-regulation of Wnt3a and Axin that were induced by FG-4592 [81]. These effects may be due to HIF-2α driven WNT5a expression [58].

There is limited work investigating HIF-2α, in vivo. HIF-2α activation in colon epithelium is essential in colon tumorigenesis in mouse models of colitis-associated colorectal cancer by mediating recruitment of neutrophils via CXCL1/CXCR2 chemokine axis [82]. This effect is also seemingly dependent on MAZ, a myc-associated Cys2-His2-type zinc finger transcription factor. Whether this axis exists in other cell types or tumor models remains unexplored. One limitation to studying transformed cells is the non-physiological activation of signaling pathways. Though we can draw information from these studies, HIFα must be further investigated in other non-transformed cellular models.

### 2.3. Epithelium

HIF-2α has also been studied in non-transformed cells. In line with previous studies in HUVECs, HIF-1α protein expression is upregulated before HIF-2α in mouse retinal organoids under hypoxic conditions [54]. HIF-1α and −2α were also shown to have redundancy in *Vegfa* mRNA expression of retinal organoids. Only siRNA targeting both was sufficient to prevent *Vegfa* mRNA expression, and VEGF protein expression was significantly decreased by either HIF-1α or HIF-2α targeted siRNA at 48 h 1% oxygen [54]. In hypoxia-treated murine bone marrow-derived macrophages (BMDMs) as well as fluorescence-activated cytometry sorted tumor CD11b+ cells, we have shown that HIF-1α or HIF-2α knockout moderately decreased *Vegfa* mRNA expression [9], suggesting partial redundancy of *Vegfa* expression by HIF-1α and −2α in TAMs.

Many pre-clinical works studying hypoxia are limited by assuming normoxia at 20% oxygen when oxygen in physiological tissues, termed physoxia, is much lower (3–9% in tissues and 1.3–2.5% cellularly). ChIP-seq analyses of HIF-1α and HIF-2α binding in HKC-8 human renal proximal tubule cells cultured in 3% versus 0.5% oxygen revealed no site-switched isoform specificity according to degree of hypoxia, suggesting that comparison of 20% oxygen to hypoxic conditions may be overexaggerating effects [43]. Even when multiple time points were introduced over 48 h, there was no evidence that sites bound specifically at one time point. Once sites were classified as HIF-1α or HIF-2α specific according to the ratio of HIF-1α to HIF-2α signal in wild-type cells, either HIF-1α or HIF-2α were knocked out in HKC-8 cells. These data showed no difference in increased binding, suggesting that HIF-1α and HIF-2α bind DNA across the genome largely independently of one another in a non-competitive and non-compensatory manner. However, HIF-1α and HIF-2α have differed distribution of binding with respect to the transcriptional sites at promoters with HIF-1α binding more frequently close to (within 5 kb) and HIF-2α binding more frequently distant (>5 kb) from transcriptional start sites in HKC-8 cells, renal cell carcinoma RCC4 cells, and hepatoma HepG2 cells [43]. This was in accordance with human MDM which showed HIF-1α bound preferentially in promoters while HIF-2α binding was more pronounced in enhancer regions [47]. HKC-8 renal RCC4 and HepG2 cell lines as well as the breast cancer cell line MCF-7 were incubated in 0.5% oxygen for 16 h and evaluated using ChIP-seq. Overall, HIF-1α sites showed a higher level of conservation among different cell lines than HIF-2α with approximately 25% of HIF-1α sites and 15% HIF-2α sites shared between two or more cell lines [43] underscoring the cell-specificity of HIF-mediated transcription.

### 2.4. Fibroblasts

Other stromal cells in the hypoxic tumor microenvironment have also provided insight into HIF-2α function in tumors. Cancer-associated fibroblast (CAF)-specific deletion of HIF-2α, but not HIF-1α, delayed pancreatic ductal adenocarcinoma (PDAC) tumor progression and growth, and improved survival of mice, suggesting a tumor promoting role for HIF-2α in fibroblasts [83]. Transcriptomic analysis revealed a stromal HIF-2α-dependent gene signature with the most notable changes being in pathways related to myeloid/macrophage biology with downregulation of genes involved in macrophage migration, differentiation, and activation including matrix metalloprotease (*Mmp9*), cluster of differentiation 74 (*Cd74*), transforming growth factor beta (*Tgfb1*), Cd11b (*Itgam)*, and complement C3a receptor 1 (*C3ar1*). CAF-specific HIF-2α-deficient tumors had a significantly lower proportion of myeloid immune cells than CAF-HIF-2α WT tumors and reduction of *Arg1*, mannose receptor C-type 1 (*Mrc1*), *Cd11b* (*Itgam*), *Cd68*, and *Adgre1* (*F4/80*) whole tumor gene expression associated with tumor promoting TAMs, suggesting that HIF-2α in CAFs indirectly modulates immunosuppressive TAMs. Moreover, immunohistochemistry (IHC) staining of tumor sections for the regulatory T cell (Treg) marker forkhead box P3 (FoxP3) showed that CAF-HIF-2α KO tumors had significantly fewer Tregs than CAF-HIF-2α WT tumors, suggesting that the survival improvement in tumor-bearing mice may have been driven by indirect deficiency of pro-tumor macrophages and T regulatory cells. Despite the advantageous effects of conditional HIF-2α KO, this effect did not translate during exogenous treatment. Treatment with HIF-2α inhibitor PT2399 had the worse survival even worse than the vehicle and IgG control [83]. This was likely due to the off-target effect of exogenous HIF-2α inhibition. Given that we have reported conditional HIF-2α deficiency in macrophages worsens murine breast tumor progression, we wonder if this effect was dependent on the macrophage population in PDAC or on another dominating cell type affected by HIF-2α inhibition [9].

### 2.5. Astrocytes

In astrocytes, like macrophages, HIF-1α and HIF-2α have seemingly divergent roles. Primary murine astrocytes respond divergently to various oxygen tensions coupled to glucose availability. For example, HIF-2α protein increased in astrocytes at 0.5% oxygen whether astrocytes were pretreated in 21% oxygen (hyperoxia) or 2% oxygen (which is much closer to physiologic oxygen tension in the brain), while HIF-1α was induced by 0.5% oxygen but was reduced to a lower level when pretreated with 2% oxygen, suggesting that HIF-2α may be more sensitive to small decreases in oxygen concentration. This sensitivity may be necessary to maintain homeostasis in the relatively immunosuppressive central nervous system environment [84]. Importantly, the authors reported that HIF-1α protein was not observed by Western blot when astrocytes were exposed to 2% oxygen over 7 days, suggesting that HIF-1α is not detected until much lower oxygen concentrations though these data were not shown. This demonstrated that HIF-2α may be induced preferentially rather than HIF-1α when exposed to acute severe hypoxia (24 h of 0.5% oxygen). In addition, astrocytes exposed to moderate 2% hypoxia and abundant glucose (10 mM) for 7 days before acute severe hypoxia (0.5% for 24 h) had increased HIF-2α and EPO (HIF-2α target gene) expression. A reduction a glucose (2 mM) in the same oxygen conditions significantly reduced HIF-2α and EPO, suggesting that glucose availability also dictates HIF mRNA and protein expression [85]. This phenomenon remains unexplored in other cell types and poses whether low glucose availability in the tumor microenvironment may dictate TAM HIF-2α expression.

### 2.6. Myeloid Cells

Perhaps the most important cell types to infer TAM HIF-2α functions are other myeloid cells. Myeloid cells include monocytes/macrophages, neutrophils, dendritic cells, macrophages, and a variety of precursors. Myeloid HIF-2α is protective in murine colitis. Myeloid knockout of HIF-1α ameliorated murine dextran sodium sulfate (DSS)-induced colitis while myeloid HIF-2α knockout aggravated colitis by increasing myeloperoxidase (MPO)+ and CD3+FoxP3+ T regulatory cell recruitment deep in the colon with no apparent differences in F4/80+ cell infiltration [86]. MPO+ cell recruitment was also increased in double myeloid knockout of HIF-1α and HIF-2α, suggesting that HIF-1α activity is not responsible for their induction, rather HIF-2α prevents their induction when both HIFα subunits are present in this model. Despite HIF-2α deficiency in all lysozyme M expressing cells (including neutrophils and monocytes/macrophages), this suggests myeloid HIF-2α in the DSS colitis model affects MPO+ neutrophil populations preferably. In immunosuppressive environments, the effects of HIFα deficiency likely dominate in macrophages as we will review later.

In neutrophils, HIF-2α gain-of-function mutations enhanced in vivo neutrophil longevity in zebrafish and reduced human neutrophil apoptosis, ex vivo [87]. However, HIF-2α knockdown in murine neutrophils showed similar apoptosis rates compared to control neutrophils, suggesting that only pathological increases in HIF-2α affect apoptosis. This is seemingly oxygen independent as freshly collected human neutrophils and cultured neutrophils express HIF-2α at normoxia and hypoxia while HIF-1α is only induced in hypoxic conditions. Murine neutrophils also have basal expression of HIF-2α [87].

Lipo-polysaccharide (LPS) induction of acute lung injury in mice deficient in myeloid HIF-2α had reduced lung injury coinciding with reduced neutrophils in bronchoalveolar lavage samples, increased neutrophil apoptosis, and no change in macrophage efferocytosis [87]. Given these models also have HIF-2α-deficient macrophages, the authors note that neutrophil accumulation could be a consequence of altered macrophage function in this model. Ex vivo studies of neutrophils suggest that they are, at least in part, responsible for these changes in the in vivo model.

Microglia, which are often referred to as resident macrophages of the brain, share common features of macrophages. One study found that blocking Cav2.2 channels of the murine microglial MG6 cell line enhanced Arg1 and IL-10 protein expression induced by IL-4, in vitro [88]. This phenomenon was dependent on HIF-2α, as HIF-C2 (HIF-2α inhibitor) prevented the upregulation of Arg-1 and IL-10. This suggests that when microglia are treated with IL-4, associated with an anti-inflammatory phenotype, that Cav2.2 may block HIF-2α-driven expression of Arg-1 and IL-10. IL-4 treatment of bone marrow-derived macrophages (BMDMs) increases *HIF-2α* and *Arg1* mRNA expression [55,89]. However, Imtiyaz et al. reported IL-4 and hypoxia alone increased BMDM arginase activity independent of HIF-2α [62].

### 2.7. Other

Studies performed in other cell types may also provide insight into possible HIF-2α targets in TAMs. For example, Koeppen et al. reported hypoxia-stabilized HIF-2α induces epithelial growth factor *amphiregulin* (*Areg*) in murine cardiac myocytes [63]. Myocyte HIF-2α-deficiency worsened myocardial ischemia-reperfusion injury in comparison to myocyte HIF-1α-deficient mice, and recombinant Areg treatment in HIF-2α-deficient mice was cardioprotective, suggesting that myocyte HIF-2α confers protection in myocardial-reperfusion injury. Meng et al. demonstrated *Areg* gene expression is significantly higher in classically activated, pro-inflammatory murine macrophages but *Areg* does not affect their pro-inflammatory cytokine production, in vitro [90]. Similarly, Lee et al. reported HIF-2α promotes transcription-independent induction of AREG receptor, epidermal growth factor receptor 1 (*ERBB1*), expression in human cardiac myocytes and demonstrated *ErbB1* expression was also cardioprotective in mice like Areg [64]. ERBBs are significantly associated with classically activated, pro-inflammatory macrophages [91]. Given this, HIF-2α-mediated *Areg* and *ERBB1* may be expressed in attempts to dampen the inflammatory response in macrophages and warrants further investigation.

## 3. Oxygen Dependent Macrophage HIF-2α Functions

Very little work differentiates HIF-1α from HIF-2α function in macrophages, and few of these works investigate hypoxia independent versus dependent functions. To understand how HIF-2α functions in TAMs, we will first review oxygen-dependent macrophage HIF-2α functions in vitro followed by review of in vivo studies in non-tumor models.

### 3.1. In Vitro

Most studies investigating macrophage HIF-2α focus on inflammatory responses. In addition to this, our laboratory is interested in macrophage HIF-mediated angiogenic regulatory functions. We previously reported local administration of a pharmacological dose of granulocyte-macrophage colony-stimulating factor (GM-CSF) induces mononuclear phagocytes to overexpress soluble VEGF receptor-1 (sVEGFR-1) and decrease vessel density in murine breast tumors [92]. Given that these tumors are hypoxic, we asked how hypoxia might drive angiogenic function in macrophages. GM-CSF induces sVEGFR-1 production from BMDMs and reduces VEGF bioactivity on endothelium [93]. We found that BMDMs deficient in HIF-1α produce significantly less VEGF [93]. When we exposed mononuclear phagocytes to hypoxia, we found that lower oxygen (0.5% 48 h) significantly enhanced GM-CSF-induced sVEGFR-1 from these cells which was abrogated by HIF-2α siRNA but not HIF-1α siRNA [93]. This indicated that HIF-2α drives sVEGFR-1under hypoxic conditions. While we demonstrated GM-CSF-induced sVEGFR-1 production was dependent on signal transducer and activator of transcription 5 (STAT5) phosphorylation and Janus Kinase 2 (JAK2) signaling as sVEGFR-1 protein induction was prevented by JAK2 inhibition via AG490, the relationship between JAK2/STAT5 and HIF-2α remain unexplored in this system.

Though previously described in vitro work in normoxic conditions suggested HIF-1α drives iNOS while HIF-2α drives *arginase 1* macrophages, hypoxic arginase1 induction after IL-4 priming is reduced in HIF-1α KO peritoneal macrophages as well just to a lesser extent than HIF-2α KO macrophages with complete reduction in double knockouts, suggesting contributions of both HIFs to *arginase 1* expression [55]. We wonder if the results would differ if the order was reversed, i.e., hypoxia priming then cytokine treatment, or if cytokines and hypoxia were given concurrently. Which treatment better reflects the tumor microenvironment? Further investigation is warranted.

In an exploratory study, HIFα binding sites and subsequent expression profiles demonstrated differential and hypoxia-dependent targets of either HIF-1α or HIF-2α in human MDMs [47]. HIF-1α bound preferentially in promoters while HIF-2α binding was more pronounced in enhancer regions. Hypoxic human MDMs analyzed by ChIP-seq reported 371 sites shared between HIF-1α and HIF-2α with 713 sites associated with HIF-1α and 795 sites for HIF-2α, suggesting that about half of all sequences are shared between the HIFα subunits. Glycolysis and gluconeogenesis pathways were most pronounced for HIF-1α target genes while peroxisome proliferator-activated receptor (PPAR) and phosphatidylinositol-4,5-bisphosphate 3-kinase (PI3K)-signaling were most pronounced for HIF-2α [47]. PCR validation confirmed ADM is regulated by HIF-2α and not HIF-1α in human macrophages [47]. IL-10 pretreatment prior to hypoxia resulted in little gene differences between HIF-1α and HIF-2α-associated genes. Of note, HIF-2α-associated matrix metalloproteinase (*MMP) 7* was enhanced with IL-10 pretreatment but the functional relevance remains unexplored.

Using HIF-1α or HIF-2α siRNA in human MDMs under hypoxic conditions successfully knocked down protein expression of the targeted HIF with no effect on the other’s expression [61]. In vitro hypoxia-mediated induction of *IL-1β* mRNA and protein expression in human MDMs was significantly decreased by miRNA knockdown of either HIF-1α, −2α, or both siRNAs. Despite studies in hypoxic HIF-2α-deficient BMDMs suggesting HIF-2α drives *Vegf, Il-1b*, and *Cxcl2* mRNA expression [62], these studies neglected comparison to HIF-1α. This may explain why Fang et al. reported miRNA knockdown of either HIF-1α, −2α, or both siRNAs significantly decreased hypoxic induction of several genes including *CXCR4, GLUT-1*, and *ADM* and CXCL8 and VEGF protein expression [61]. In contrast, *ADORA2A* mRNA expression was only downregulated by HIF-2α siRNA in human MDMs. This agreed with hypoxia-treated murine BMDMs from myeloid HIF-1α-deficient mice which has unchanged *ADORA2A* gene expression when compared to BMDMs from control mice. Primary human macrophages transfected with siRNA targeting HIF-2α expression prevented hypoxic induction (1% for 48 h) of *CTSB* (Cathepsin B) and *SNX5* (sorting nexin 5) mRNA [60]. However, the functional relevance of these genes was unexplored. It is likely hypoxia in combination with other signals are required for robust gene expression, in vitro.

Despite redundancy of HIFα-mediated expression of *ADM, VEGF*, and *IL-1b* in human MDMs, murine BMDMs deficient in HIF-2α have reduced expression of those genes as well as *Cxcl2* mRNA expression in hypoxic (0.5%) BMDMs. When combined with LPS and IFNγ treatment, hypoxia treated myeloid HIF-2α-deficient BMDMs reduced induction of *Il1b, Il12, Cxcl2, Vegf, Adm*, and *Il6* mRNA but had no effect on nitrate or iNOS expression in addition to MHC class II expression [62]. Importantly, this study demonstrated primary myeloid cells that experience HIF-2α deletion in this mouse model (LysMcre/HIF2α*^flox/flox^*) are cells of the monocyte/macrophage lineage reporting no changes in bone marrow myeloid progenitor populations, macrophage maturation, and neutrophil populations/differentiation [62].

Interestingly, LPS and IFNγ-treated BMDMs had no difference in NO levels between control and HIF-2α-deficient BMDMs in normoxia or hypoxia which was attributed to different mouse strains and treatment conditions [62]. We have also produced similar results which demonstrated iNOS protein expression is unchanged in IFNγ/LPS-treated HIF-2α-deficient BMDMs in normoxic or hypoxic conditions while iNOS expression was reduced in HIF-1α-deficient BMDMs regardless of oxygen conditions, suggesting that HIF-1α confers iNOS expression in acute inflammation settings. Imitiyaz et al. suggested mixed 129/Sv x C57BL/6J genetic background possibly alters the penetrance of HIF-2α mutant phenotypes as hypoxic phenotypes were observed consistently in 70% of the myeloid HIF-2α-deficient mice. Additionally, this group used heterozygous BMDMs as controls, suggesting that HIF-2α effects may be more pronounced than what was investigated.

### 3.2. In Vivo

**Figure 3.**
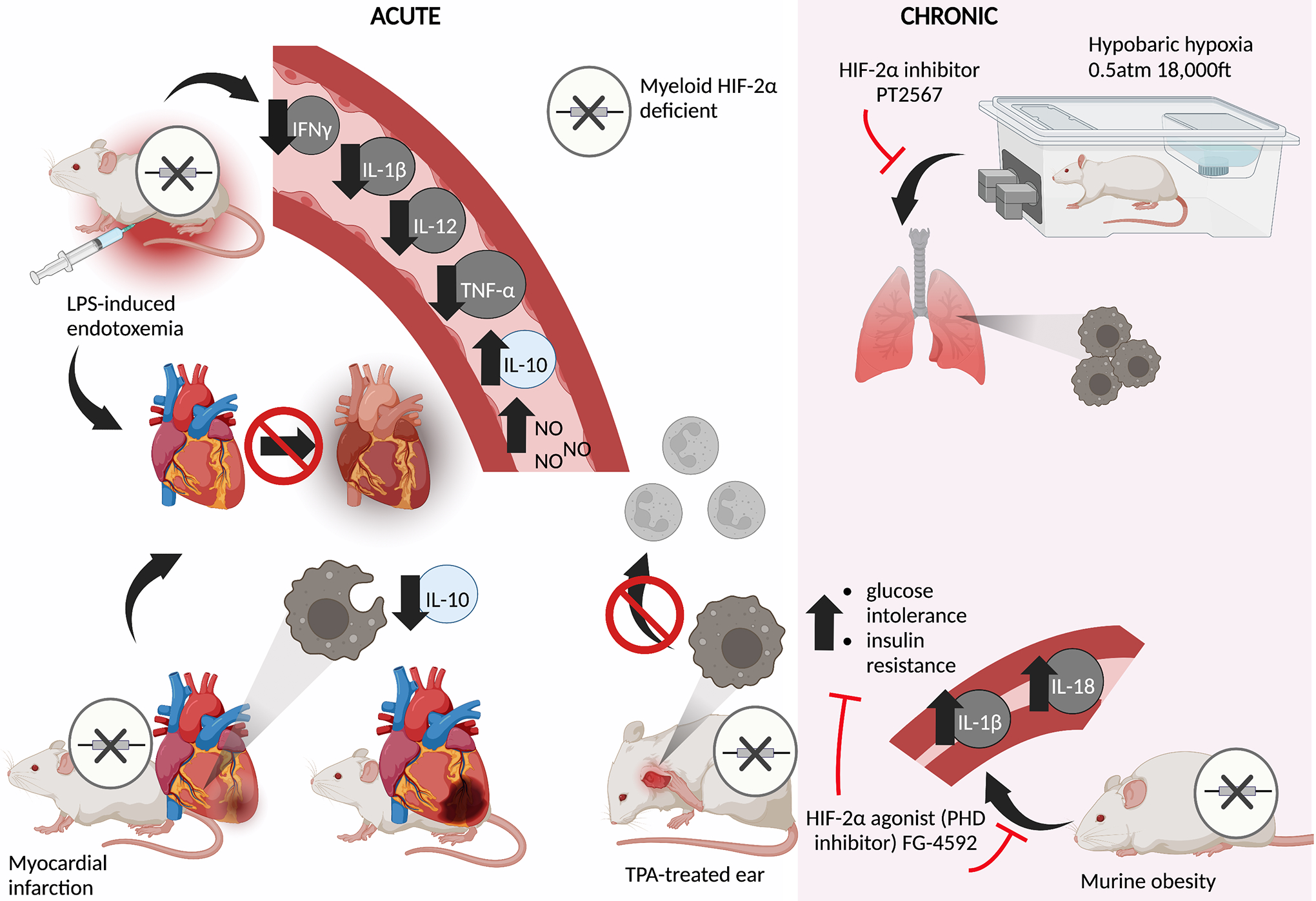
TAM HIF-2α functions. Macrophage HIF-2α has been investigated in several tumor models (individual, colored bubbles). Myeloid HIF-2α deficiency increases tumor foci in a Lewis lung cancer (LLC) extravasation model and worsens survival in fibrosarcoma-bearing mice. Other studies suggest overlapping functions (large, overlapping bubbles). HIF-2α agonism with synergistic treatment with local GM-CSF slows melanoma growth and promotes the production of macrophage-derived sVEGFR-1 to dampen excessive angiogenesis. Myeloid HIF-2α deficiency abrogates macrophage-derived sVEGFR-1 effects and increases melanoma-specific *Pmel17* mRNA in lungs of melanoma-bearing mice. In breast tumor-bearing mice, myeloid HIF-2α deficiency increases vessel density and reduces tissue oxygenation. HIF-2α inhibition significantly reduces tumor weight, VEGF production, vessel density, and whole tumor *Mrc1* mRNA expression in subcutaneous LLC tumors. In another breast tumor model, macrophage HIF-2α drives Spint1 tumor suppressor expression which inhibits in vitro breast cancer cell growth. Myeloid HIF-2α deficiency results in faster orthotopic breast tumor growth, tumor IL-10 reduction, and decreased TAMs at end point. A reduction of TAMs was also seen in inflammation-induced hepatocellular carcinoma (HCC) and colon carcinoma of myeloid HIF-2α-deficient mice.

Few studies have investigated HIF-2α functions in TAMs. We have shown that myeloid HIF-2α modulates HIF-1α driven proangiogenic responses by expressing sVEGFR-1, stabilizing proliferating vessels, and promoting healthy revascularization in murine melanoma (Figure 3) [94]. In vivo treatment of melanoma-bearing mice with pharmacological doses of GM-CSF inhibits tumor growth. This effect was dependent on macrophage HIF-2α. Myeloid HIF-2α deficiency also led to increased expression of melanoma-specific *Pmel17* mRNA in lungs of tumor-bearing mice, suggesting a protective role of myeloid HIF-2α in the spread of murine melanoma [94]. In a follow-up study, we found HIF-2α stabilization by Akebia Therapeutics (AKB)-6899 decreased tumor growth and improved survival [71] with a moderate decrease in vessel density. We now believe these tumors likely have better perfusion which may not be adequately reflected by endothelial cell density measurements alone. In addition, tumor volumes of human melanoma xenografts in severe combined immunodeficiency (SCID) mice had only a marginal, non-significant response to AKB-6899 alone, suggesting that HIF-2α activation in immune cells, particularly macrophages, is likely required for the decreased tumor growth and improved survival seen in immunocompetent mice. To our knowledge, this study served as the first demonstrating activation of a HIF protein could decrease tumor growth. Another interesting finding in this work was that HIF-2α stabilization with AKB-6899 alone had no effect on the number of CD68+ macrophages infiltrating into the melanoma tumor, suggesting that the macrophage phenotype is likely responsible for the difference seen at this time point. This hypothesis is further supported by our recent work demonstrating myeloid HIF-2α deficiency also had no effect on F4/80+ macrophages in murine breast tumors despite significant phenotype differences at that time point [9]. Myeloid HIF-2α deficiency also accelerated tumor development in a murine fibrosarcoma model, suggesting a tumor-repressing ability of myeloid HIF-2α [95].

Other tumor studies suggest that macrophage HIF-2α may be detrimental. For example, a study from Leek et al. suggests that HIF-2α accumulation in tumor-associated macrophages from patients with breast cancer correlate with high microvessel density and tumor grade [96], and Liu et al. reported TAM HIF-2α in human lung adenocarcinoma correlates with worse survival though HIF-1α was not investigated [97]. However, mice deficient in myeloid HIF-2α had breast tumors with increased microvessel density and lower oxygen tension underscoring that vessel density does not equate vessel perfusion [9]. It is likely that the correlation found between HIF-2α expression in TAMs with increased vessel density in human breast tumors was confounded the hypoxic nature of the tumor microenvironment and may have been explained by concurrent HIF-1α as our work suggests myeloid HIF-2α expression reduces murine breast tumor vascularity.

In murine hepatocellular and colitis-associated colon carcinoma models, macrophage HIF-2α deficiency revealed that HIF-2α regulates tumor macrophage recruitment and suggested that it was due to *Csf1* and *Cxcr4* mRNA expression using in vitro studies [62]. The number of TAMs recruited to liver tumors was reduced in myeloid HIF-2α-deficient mice in comparison to controls and correlated with a reduction of high-grade tumors. In the colitis-associated colon carcinoma model, TAM infiltration was also reduced, and HIF-2α deficiency resulted in a marked, yet insignificant, reduction in tumor burden. These findings contrast an E0771 allograft breast tumor model which revealed faster growth in myeloid HIF-2α-deficient mice [59] as well as our orthotopic Py8119 breast tumor model which has significantly larger tumors when myeloid cells are deficient in HIF-2α and the infiltration of F4/80+ macrophages were unchanged [9]. The percentage of myeloid-derived (neutrophils, monocytes, macrophages, dendritic cells) nor lymphoid cells (NK cells, B cells, T cells) between control and myeloid HIF-2α-deficient tumors using the E0771 orthotopic murine breast tumor model were also unchanged when tumor burden became significantly different (~13 days) nor at end point (~16 days/1.2 cm) [59]. Extending our murine breast tumor model (>28 days/2 cm), we found a significant reduction in tumor macrophages in myeloid HIF-2α-deficient mice compared to controls, suggesting that reduced TAMs from myeloid HIF-2α deficiency may span several tumor types but the length of tumor progression matters (results in preparation for publication). Whether less TAMs are recruited to the tumor or TAMs are lost more rapidly in myeloid HIF-2α-deficient mice is yet to be determined. Likely, the differences in tumor burden across models are different because tumorigenesis in the orthotopic murine breast tumor models are significantly different from inflammation-associated autochthonous hepatocellular and colitis-associated colon carcinoma models.

At end point using the E0771 orthotopic murine breast tumor model (1.2 cm), differential gene expression by RNA-sequencing of wild-type versus HIF-2α-deficient TAMs demonstrated 3 genes: *Spint1, IL-10*, and *Depdc7* were downregulated by HIF-2α knockout and had an identified HIF-2α (not HIF-1α) binding site using previously published ChIP-seq data sets [47]. Spint1 is a tumor suppressor and recombinant Spint1 inhibited E0771 breast tumor cell proliferation, in vitro. A variety of soluble mediators including CXCL1, CCL2, TNFα, IFNγ, IL-1β, IL-2, IL-4, and IL-6 were unchanged. Only IL-10 was significantly reduced in myeloid HIF-2α-deficient tumors at end point. Though IL-10 has been largely associated with immune cell suppression and particular suppression of cytotoxic T cells [98], more recent work suggests IL-10 can expand terminally exhausted CD8+ tumor infiltrating lymphocytes and promote their effector function leading to eradication of pre-clinical models of solid tumors [99,100]. Perhaps myeloid HIF-2α drives excessive IL-10 in a tumor suppressive mechanism such as this.

TAMs from subcutaneous Lewis lung carcinoma (LLC) had higher HIF-2α protein expression in *Atp6v0d2*-deficient mice, suggesting that ATP6V0d2, a proton-transporting ATPase, suppresses HIF-2α protein. Specifically, ATP6V0d2 mediates lysosomal degradation of HIF-2α in macrophages limiting its expression [97]. HIF-2α inhibition using PT2385 significantly reduced tumor weight, VEGF production, vessel density, and whole tumor Mrc1 expression while increasing pericyte-coated vessels in subcutaneous LLC tumors [97], though Lu et al. reported increased LLC tumor foci in lungs of myeloid HIF-2α deficient mice using an extravasation model [101]. The divergence of LLC results is likely due to the differences in subcutaneous and lung tumors. We also found a significant reduction in M2-like *Mrc1* mRNA expression in our studies using orthotopic breast tumors in myeloid HIF-2α-deficient mice but significant increases in M2-like *Lyve1* and *Ym1*, suggesting that conclusions of myeloid HIF-2α driving alternative activation in TAMs require further investigation (data not published). We have also reported that orthotopic breast tumors in myeloid HIF-2α-deficient mice have higher pericyte coverage; however, this was accompanied with decreased vessel perfusion, exacerbated hypoxia, and increased tumor burden in our model that may be explained by tissue specific differences [9].

Despite the likelihood of these solid tumor models having a heterogeneity of functional vessels and hypoxia, to our knowledge we are the only group that has investigated myeloid HIF-2α function and the concentration of oxygen in the hypoxic tumor concurrently [9]. Clearly, more work must be done to discern the hypoxia independent and dependent functions of HIF-2α in TAMs.

## 4. Oxygen Independent Macrophage HIF-2α Functions

### 4.1. Non-Human Studies

Our laboratory previously reported that, AKB-6899, a small molecule inhibitor of PHD3, stabilizes HIF-2α specifically even at normoxia while leaving HIF-1α susceptible to proteosome degradation to prolyl hydroxylation by PHD2 in murine BMDMs and acts synergistically to produce the anti-angiogenic sVEGFR-1 in response to treatment with GM-CSF [71]. The addition of GM-CSF in combination with AKB-6899 revealed synergy with the production of sVEGFR-1 significantly higher in the combination treatment than GM-CSF alone independent of oxygen. In fact, hypoxia or HIF-2α stabilization by AKB-6899 had similar BMDM production of sVEGFR-1, suggesting that HIF-2α stabilization regardless of hypoxia is responsible for these findings. The effect of AKB-6899 on sVEGFR-1 production was completely lost in BMDMs deficient in HIF-2α (LysMcre/HIF-2α*^flox/flox^* mice) but not in BMDMs deficient in HIF-1α (LysMcre HIF-1α*^flox/flox^* mice), or by using a PHD2-specific inhibitor, AKB-4924, indicating the dependency on HIF-2α [71].

Other work has focused on relating HIFα signaling to activation classification of macrophages. Briefly, macrophages have been generally categorized as classically activated (M1) versus alternatively activated (M2) based on responses involved in type I helper T cell (Th1) responses and Th2 responses, respectively. Interferon gamma (IFN-γ) and toll-like receptor engagement generate M1-like macrophages associated with pro-inflammatory responses with increases in major histocompatibility complex (MHC) class II, IL-12, and nitric oxide (NO) generation, while IL-4 and IL-13 generate M2-like macrophages which are associated with inflammatory resolution and oppose changes observed in their M1-like counterparts [102]. The cancer immunology field now recognizes that TAMs do not follow this strict M1/M2 definition in solid tumors and instead share features of both [103]. Ma et al. have recently used single cell multi-omic technologies to cluster TAM subsets in an unbiased manner [104]. TAMs are more aptly categorized into seven groups based on transcription signatures: interferon-primed TAMs, immune regulatory TAMs, inflammatory cytokine-enriched TAMs, lipid-associated TAMs, pro-angiogenic TAMs, resident-tissue macrophage-like TAMs, and proliferating TAMs. These major TAM subsets represent an improved guideline to characterize TAM diversity. For clarity, we will continue to use the M1/M2 nomenclature to describe previous work but note its limitation and suggest future work adopt this more diverse classification of TAMs.

Perhaps one of the most formative studies demonstrated that *Hif1a* mRNA expression increased in IFNγ- or LPS-treated BMDMs over 24 h peaking at 12 h (significant at 6, 12, 24 h) while repressing *Epas1* (HIF-2α) mRNA expression relative to controls [55]. LPS- and IFNγ-induced HIF-1α protein in normoxic (21%) conditions while HIF-2α protein was largely undetected in normoxic conditions despite cytokine pre-treatment, suggesting that M1-primed macrophages may have preferential HIF-1α expression. Matak et al. demonstrated this effect persists over 48 h of IFNγ treatment [89]. In contrast, IL-4 treatment of BMDMs increased HIF-2α (highest at 48 h), *Fn1*, and *Arg1* mRNA expression over 48 h with no effect on *HIF-1α* mRNA expression, suggesting that alternately activated macrophages may have preferential activation of HIF-2α [55,89]. Importantly, these data were produced independent of hypoxia.

For comparison, thioglycolate-induced peritoneal macrophages were subjected to like conditions. Similarly, peritoneal macrophages under normoxic conditions had detectable HIF-2α protein at baseline that decreased with LPS or IFNγ pre-treatment and increased with IL-4 treatment [55]. These effects were accentuated after 4 h of hypoxia but was not dependent on hypoxia. However, Imtiyaz et al. reported IL-4 and hypoxia alone increased BMDM arginase 1 activity, but this was not dependent on HIF-2α [62]. HIF-2α was also not required for MRC1 expression which is associated with the M2-like anti-inflammatory macrophage phenotype, suggesting that M2-priming of macrophages may increase HIF-2α but HIF-2α does not confer the M2-like phenotype [62].

Yet, other studies suggest HIF-2α may contribute to a M2-like phenotype. Peritoneal macrophages from HIF-2α*^flox/flox^*;*Tek*cre+/− showed loss of HIF-2α did not affect M2 gene expression of *Fizz1* and *Ym1* but did decrease *arginase1* expression, a canonical M2 marker, in normoxic conditions [55]. Because iNos and arginase1 were used to classify M1 versus M2 polarized macrophages at this time, the authors investigated their mRNA expression in peritoneal macrophages from HIF-1α*^flox/flox^*;*Tek*cre+/− and HIF-2α*^flox/flox^*;*Tek*cre+/− mice. *iNos* was reduced in HIF-1α-deficient peritoneal macrophages but not in those deficient in HIF-2α, suggesting that *iNos* induction is dependent on HIF-1α, and HIF-1α may be associated with a pro-inflammatory macrophage phenotype. HIF-2α knockdown using siRNA in peritoneal macrophages decreases *Arg1* mRNA expression and increases NO production upon palmitate challenge, suggesting that HIF-2α would normally suppress NO production upon challenge [56]. Chronic palmitate treatment of BMDMs has been associated with M2 polarization [105]. In addition, HIF-2α knockdown exacerbated induction of *Tnfa* and *Il-6* mRNA expression, again only after palmitate challenge [56].

Constitutive expression of myeloid HIF-1α (myeloid Vhl mutant/HIF-2α knockout) in LPS-treated murine peritoneal macrophages increased *iNos* mRNA expression further supporting HIF-2α suppressing HIF-1α-mediated *iNos* expression. In contrast, constitutive expression of myeloid HIF-2α (myeloid *Vhl* mutant/HIF-1α knockout) in IL-13-treated murine peritoneal macrophages increased *Arg1* mRNA expression, suggesting that HIF-1α may suppress HIF-2α-mediated *Arg1* expression [106].

Susen et al.’s investigation of the classic M1 versus M2 phenotype did not support the divergence in macrophage HIF-1α and HIF-2α inflammatory phenotype at baseline. For example, there was no difference in surface expression of polarization markers CD80 (M1-like) and CD206 (M2-like) nor *Il1b, iNos, Tnfα, Arg-1, Tgfb*, and *Fizz1* mRNA expression between wild-type and HIF-2α-deficient BMDMs [59]. This was likely due to the unpolarized quiescent status of BMDMs in this study. Together, these data suggest that inflammatory polarization signals are required for HIFα effects in macrophages.

Only a few other studies demonstrate oxygen-independent macrophage HIF-2α functions unrelated to angiogenic responses or macrophage polarization. Susen et al. reported HIF-2α-deficient BMDMs produce less serine peptidase inhibitor Spint1 (tumor suppressor) than controls regardless of oxygen concentration resulting in faster orthotopic breast tumor growth, suggesting a paracrine role for HIF-2α in tumor suppression [59]. BMDMs regardless of hypoxic conditions also have reduced in vitro migration and invasion to chemoattractant colony stimulating factor1 (CSF1) when derived from myeloid HIF-2α-deficient mice suggesting a role for HIF-2α in macrophage recruitment [62]. Similarly, chemoattractant CSF1 and pro-inflammatory cytokine IL-6 enhance macrophage HIF-2α, but not HIF-1α, protein expression through PPARγ under normoxic conditions perhaps driving migration and invasion of macrophages [107].

HIF-2α protein expression is increased in TAMs from HCC murine tumors [108]. However, TAM *Hif-2α* mRNA expression was not different from non-tumor-infiltrating macrophages, suggesting posttranscriptional regulation of HIF-2α in TAMs [108]. Evidence in human ECs suggests increased stability of HIF-2α protein compared to HIF-1α due to the *EPAS1* (HIF-2α) 3′ UTR being less prone to ARE-dependent destabilization than HIF1A mRNA [40]. Instead, this group investigated endogenous miRNA targeting. In vitro work suggested that HIF-2α is suppressed at 3′UTR by miR-17 and miR-20a and treatment with tumor cell supernatant or autocrine-derived IL-6 abrogates miR-17 and miR-20a-mediated suppression of HIF-2α in healthy peripheral blood monocyte-derived macrophages in an oxygen-independent manner suggesting, despite being a pro-inflammatory cytokine, that pleiotropic IL-6 can preferentially induce macrophage HIF-2α, indirectly [108]. Other soluble factors in the tumor microenvironment may also relieve suppression of HIF-2α functions despite tumor oxygen concentrations.

### 4.2. Human Studies

Few studies have investigated HIF-2α-mediated gene expression in human macrophages. Twenty years ago, macrophages in human ovarian, breast, and prostate tumors were found to express abundant HIF-1α, while HIF-2α was not investigated. Given this, it was hypothesized that HIF-1α may be the major HIFα in macrophages [109]. MDMs and the human macrophage-like cell line MonoMac6 also accumulate higher levels of HIF-1α than HIF-2α when exposed to hypoxia, in vitro (0.5% or 0.1% oxygen for 16 h) further supporting this hypothesis [109]. However, HIF-2α expression has also been found in subsets of CD68+ TAMs in human tumors (bladder, brain, breast colon, liver, lung, ovarian, prostate, PDAC) in addition to subsets of bone marrow macrophages [110]. Though HIF-2α may not be the highest expressed HIFα in macrophages, its presence in human tumors led to several investigations focused on explaining its function.

In one seminal study, Elbargbati et al. exposed human monocytes and MDMs were exposed to hypoxic (0.1% over 24 h) and re-oxygenation conditions [44]. There were significant differences in temporal expression between HIF-1α and HIF-2α in macrophages while monocytes did not have an induction of either HIF-1α or HIF-2α. HIF-1α protein was detected in MDM starting at 1 h of hypoxia, increased over 6 h before peaking at 16 h and then declined by 24 h. HIF-2α protein was rapidly detected by 1 h of hypoxia and maintained at 6 h before slight decline at 16 and 24 h, suggesting a possible preferential switch from HIF-1α to HIF-2α in chronic hypoxia. Following 16 h of hypoxia, MDMs were returned to normoxia (20.9%). While HIF-1α expression rapidly degraded with re-oxygenation, HIF-2α remained elevated 2 h after re-oxygenation, suggesting that HIF-2α may be more stable [44].

For gene expression in hypoxia-treated human macrophages, Tausendschon et al. reported *ADM* is regulated by HIF-2α and not HIF-1α [47]. There is a severe deficiency in human macrophage HIF-2α-driven gene expression data available in the literature and further investigation is warranted. Other studies focus solely on the presence of macrophage HIF-2α and its correlation with protein expression in human tumors. For example, high HIF-2α protein expression in tumor-associated macrophages has been correlated with increased tumor microvessel density, decreased thymidine phosphorylase, and advanced tumor grade in human breast cancer [96]. HIF-2α and CD68+ tumor-associated macrophages resided in areas with high VEGF in the perivascular niche of neuroblastoma tumors, suggesting that they may be facilitating angiogenesis at these sites [111]. Additionally, MDMs treated with tumor cell line supernatants had increased HIF-2α expression at normoxia leading to *VEGFA* and *PDGFB* transcription, suggesting an oxygen-independent pro-angiogenic response [108]. However, it is unclear if macrophage HIF-2α is driving a pro-angiogenic program in human tumors or if HIF-2α is being upregulated in response to excessive angiogenesis requirements. Our work demonstrating murine macrophage HIF-2α is responsible for sVEGFR-1 expression and VEGF sequestration suggests the latter [93].

## 5. Acute Models

Most research considering myeloid HIF expression focuses on simultaneous expression or deletion of both HIF-1α and HIF-2α subunits. Stabilization of both myeloid HIF-1α and −2α by *Vhl* deletion promoted pro-angiogenic markers *Vegf* and *bFgf* mRNA expression in CD11b+ cells and enhanced central retinal vascular regeneration with increased neovasculature in mice subjected to oxygen-induced retinopathy (OIR) [112]. This is not surprising given that both HIFs are implicated in neovascularization. There was significantly less retinal CD11b+ cells from myeloid *Vhl* mutated mice (HIF-1α and −2α constitutive expression) in the neovascular area compared to floxed littermates following OIR, suggesting that myeloid HIF-1α and −2α co-expression may affect trafficking of CD11b+ cells to the retinas, but their relative contributions were unexplored [112].

Another acute inflammatory process of interest involving myeloid cells is the activation of the inflammasome. LPS-primed BMDMs from wild-type and myeloid HIF-2α-deficient mice were treated with inflammasome activators [113]. In response to nigericin, BMDMs from myeloid HIF-2α-deficient mice had higher IL-1β and IL-18 secretion while TNF-α was unchanged suggesting that myeloid HIF-2α may suppress inflammatory activity. This effect was lost upon treatment with Nlrp3 (inflammasome sensor protein) siRNA and could be recapitulated using human MDMs. In contrast, no differences in cytokines were detected in response to muramyl dipeptide, flagellin, or poly (dA;dT) indicating specificity to inflammasome activation. LPS and nigericin stimulated BMDMs from myeloid HIF-2α-deficient mice also had greater formation of the apoptosis-associated speck-like protein containing a caspase recruitment domain (ASC) which is required for NLRP3-dependent activation of caspase-1. In the same study, BMDMs from myeloid HIF-2α-deficient mice had higher oxygen consumption rate (OCR), basal OCR, spare respiratory capacity (SPC), maximal OCR, and OCR/extracellular acidification rate (ECAR) as well as higher fatty acid oxidation enzyme carnitine palmitoyltransferase 1A (CPT1A) protein expression and activity upon LPS and nigericin stimulation compared to control mouse BMDMs, suggesting that macrophage HIF-2α suppresses OCR indicative of fatty acid oxidation during NLRP3 inflammasome activation [113]. The data suggested that macrophage HIF-2α inhibits CPT1-A mediated fatty acid oxidation to prevent excessive activation of the NLRP3 inflammasome. Interestingly, this study also suggested that HIF-2α does not suppress translation of Cpt1a by binding at the promoter but rather depleted one-carbon-unit S-adenosylmethionine (SAM) and increased enrichment of H3K27me3 histone methylation in the Cpt1a promoter to negatively regulate NLRP3 inflammasome activation [113]. LPS-induced endotoxemia in mice revealed myeloid HIF-2α also reduced the induction of proinflammatory serum levels of IL-1β, IL-12, TNF-α, and IFN-γ. Myeloid HIF-2α deficiency exacerbated induction of anti-inflammatory IL-10 in the serum in the endotoxemia model. These mice also demonstrated some protection from LPS-induced cardiac impairment which is in line with a study investigating myocardial infarction [62].

**Figure 4.**
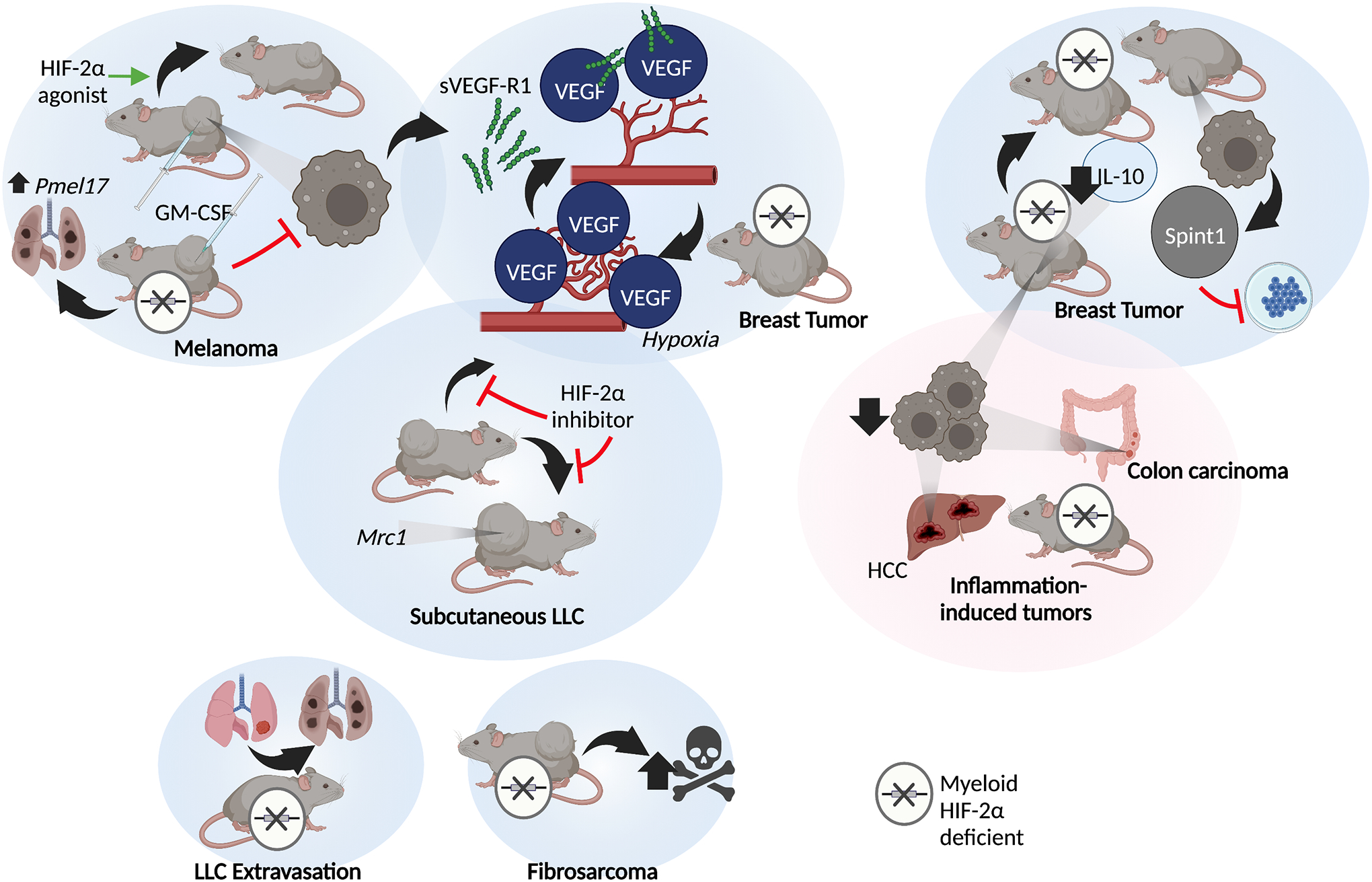
Macrophage HIF-2α function in acute vs. chronic non-tumor models. LPS-induced endotoxemia in myeloid HIF-2α-deficient mice reduces systemic levels of pro-inflammatory cytokines and increases IL-10 and nitric oxide (NO) in circulation while also reducing cardiac damage. Myocardial infarction in myeloid HIF-2α-deficient mice reduces IL-10 production by cardiac macrophages. Myeloid HIF-2α reduces macrophage-mediated neutrophil recruitment in TPA-treated ears. HIF-2α inhibition inhibits macrophage lung infiltration in rats in chronic hypoxia conditions. Systemic increases in pro-inflammatory cytokines, glucose intolerance, and insulin resistance in myeloid HIF-2α-deficient mice fed a high fat diet is abrogated by HIF-2α agonism with PHD inhibitor FG-4592.

Furthermore, in vivo treatment of mice with LPS demonstrated that plasma NO was reduced at 6 h in myeloid specific HIF-1α knockout mice and was increased at 24 h in myeloid specific HIF-2α knockout mice compared to controls suggesting that HIF-1α drives NO production by inducing *iNos* while HIF-2α suppresses NO production by competitive usage of L-arginine away from HIF-1α-driven iNOS-generated NO (Figure 4). Macrophage that preferentially induce iNOS or arginase have been called M1 and M2, respectively. This nomenclature was introduced based on C57BL/6J mouse macrophages (M1 polarized) were more easily activated to produce NO than macrophages from Balb/c mice (M2 polarized) [114]. Mantovani et al. then expanded this nomenclature to in vitro activated macrophages: M1 for macrophages treated with INF-γ/LPS or TNF, M2a for macrophages treated with IL-4, M2b for macrophages induced by Fc receptor engagement by immune-complexes and M2c for macrophages treated with IL-10 and glucocorticoids [115]. The current field recognizes classifying macrophage polarization using iNOS and arginase1 is grossly oversimplified and macrophage phenotype lies on a spectrum. Macrophages are notably plastic and instead have a diversity of subtypes that do not fit this binary classification [116]. Unfortunately, especially for M2 gene signatures which are used to describe TAMs, in vivo and in vitro comparison of macrophage transcriptional profiles have significant deviations [117]. New classifications by Ma et al. using advanced multi-omic technologies to describe transcription signatures better describe TAM signatures, and we suggest adopting this classification in future work [104]. Nonetheless, these works were first to find the diverging roles for macrophage HIF-1α and HIF-2α. This HIF-1α function was not surprising given that HIF-1α had been linked to inflammatory responses and antimicrobial activities in myeloid cells prior [118,119]. However, the HIF-2α function demonstrated here was the first to suggest a suppressive role of HIF-2α on HIF-1α in macrophages.

Despite myeloid HIF-2α-mediated inflammatory suppression in vitro, the inflammatory function of myeloid HIF-2α in vivo is complex. Recently, a murine model of myocardial infarction (MI) was used to examine proinflammatory roles for myeloid HIF-1α and HIF-2α. Increases in macrophage HIF-2α were more acutely responsive to ischemic insult and proceeded increases in HIF-1α expression [120]. Myeloid HIF-2α deficiency (LysMcre/HIF-2α*^flox/flox^*) resulted in smaller sized infarcts, improved left ventricular (LV) systolic function, reduced LV dilatation, and increased LV wall thickness, suggesting a detrimental role for myeloid HIF-2α in MI. HIF-2α deficiency did not affect the levels of HIF-1α in cardiac macrophages, suggesting that the effects were not due to compensatory effects of HIF-1α. In vitro work suggested HIF-2α activated fatty acid synthesis to antagonize CPT1-dependent fatty acid oxidation of apoptotic cell-derived fatty acids and impaired inflammatory production of IL-10 from efferocytic macrophages [120]. Despite HIF-2α driving anti-inflammatory macrophage mitochondrial metabolism in vitro, these data suggest a pathological role for HIF-2α in myeloid cells after myocardial infarction [120].

Given the LysMcre/HIF-2α*^flox/flox^* murine mouse model depletes HIF-2α in neutrophils and macrophages, several works have investigated multiple myeloid cell types. Acute cutaneous inflammation by painting mouse ear skin with 12-O-tetradecanoylphorbol-13-acetate (TPA) demonstrated a reduction in the number of neutrophils infiltrating TPA-treated ears in myeloid HIF-2α-deficient mice which was attributed to a suspected reduction in neutrophil chemokines produced by macrophages as HIF-2α was not detected in neutrophils [62].

## 6. Chronic Models

Non-tumor chronic models may better reflect the possible functions of HIF-2α in TAMs exposed to chronic hypoxia and inflammation in solid tumors. Rats housed in hypoxia for four days show increased pulmonary infiltration of ED1 + cells (rat macrophage marker) in immunohistochemical images of lung vasculature which was attenuated qualitatively by the HIF-2α inhibitor PT2567 [52]. Given the non-specific cell targeting of PT2567, it is unclear if loss of macrophage infiltration was due to direct macrophage HIF-2α inhibition or if HIF-2α inhibition indirectly decreased macrophage infiltration through the suppressed recruitment functions of another cell type.

In another chronic mouse model, mice on a high fat diet for 6 weeks had decreased HIF-2α expression in macrophages from visceral adipose tissue [113]. In mice deficient of myeloid HIF-2α fed a high fat diet for 8 weeks, glucose intolerance and insulin resistance were exacerbated when compared to control mice. Plasma levels of IL-1β and IL-18 were also significantly higher in the myeloid HIF-2α-deficient mice, suggesting that myeloid HIF-2α may suppress systemic inflammation in this model. When mice on high fat diet were treated with HIF-2α agonist FG-4592 (prolyl hydroxylase inhibitor), impaired glucose tolerance and insulin resistance was alleviated with no effect on fasting glucose or body weight and significantly reduced serum IL-1β and IL-18 [113]. This contrasted with an acute LPS-endotoxemia model demonstrating BMDMs with myeloid HIF-2α deficiency reduced hypoxia-induced (0.5%) production of proinflammatory (*Il1b, Cxcl2*) mRNA [62], suggesting that myeloid HIF-2α function varies considerably from acute to chronic inflammatory models. Given that FG-4592 also stabilizes HIF-1α [121,122], it is possible that studies using FG-4592 reflect HIF-1α function that may dominate over HIF-2α.

Choe et al. also demonstrated that elevated macrophage HIF-2α attenuates adipose tissue inflammation and improves insulin resistance in a murine obesity model [56]. *Hif-2α* mRNA expression was significantly higher in M2-like adipose tissue macrophages (ATMs) (CD11b+F4/80+CD11c-CD206+) than M1-like ATMs (CD11b+F4/80+CD11c+ CD206-) while HIF-1α was significantly lower in M2-like ATMs suggesting that HIF-2α may be more associated with immunosuppressive phenotype in macrophages. Whether alternative activation increases HIF-2α expression or vice versa has not been elucidated. Global heterozygous knockdown of HIF-2α aggravated insulin resistance and has more pro-inflammatory ATMs compared to wild-type mice which was relieved by macrophage depletion suggesting that the increase in proinflammatory ATMs may be responsible for the insulin resistance upon high fat diet. The aggravated insulin resistance reported in HIF-2α knockdown mice may be due to unchecked HIF-1α-driven adipose dysfunction, as myeloid cell-specific deletion of HIF-1α protects against high-fat diet-induced adipose tissue dysfunction [123]. Perhaps macrophage HIF-2α suppresses the negative effects of macrophage HIF-1α in this model.

Lastly, mice exposed to 5 weeks of severe intermittent hypoxia which may better reflect the varying perfusion experienced in solid tumors, wound-associated macrophages had a significant reduction in *Hif-2α, Cd206*, and *Vegfa* gene expression, suggesting that chronic intermittent hypoxia may shift macrophage function away from Hif-2α mediated effects. However, HIF-1α was not investigated thus these effects cannot be linked to HIF-2α expression alone [124].

## 7. Targeting HIF-2α Clinically

With so little work in pre-clinical tumor models, there are limited data targeting HIF-2α clinically, especially in cancer patients. Yet, there is merit in promoting macrophage HIF-2α stabilization based on the reviewed pre-clinical studies. For example, myeloid HIF-2α promotes sVEGFR-1 expression, vessel stabilization, and healthy revascularization in murine melanoma [94]. Myeloid HIF-2α deficiency also led to increased expression of melanoma-specific *Pmel17* mRNA in lungs of tumor bearing mice, suggesting a protective role of myeloid HIF-2α in the spread of murine melanoma [94]. In addition, HIF-2α stabilization by AKB-6899 decreased tumor growth and improved survival in melanoma-bearing mice [71]. Myeloid HIF-2α deficiency also accelerated tumor development in a murine fibrosarcoma model suggest a tumor-repressing ability of myeloid HIF-2α [95]. Similarly, an E0771 allograft breast tumor model revealed faster growth in myeloid HIF-2α-deficient mice [59] as well as in our orthotopic Py8119 breast tumor model [9]. Lu et al. reported increased LLC tumor foci in lungs of myeloid HIF-2α-deficient mice using an extravasation model, suggesting that macrophage HIF-2α may prevent metastatic seeding [101]. Given the growing body of pre-clinical data on macrophage HIF-2α, understanding the feasibility of targeting HIF-2α in TAMs in patients is necessary for therapeutic advancement.

Currently, no clinical trial is testing HIF-2α agonism for therapeutic treatment of cancer patients. Instead, HIF-2α activation has emerged as a renal anemia treatment. Recently, a benzisothiazole derivative has been identified as a HIF-2α agonist [125]. Its combination with prolyl hydroxylase inhibitor AKB-6548 (vadadustat) synergistically increases plasma erythropoietin level in mice [126]. Although AKB-6548 may induce HIF-2α-dependent gene expression with little effect on HIF-1α-dependent genes such as VEGF [71], other studies suggest PHD inhibitors increase the levels of the three HIF subtypes with poor selectivity for HIF-2α [127]. So far, more than 10 PHD inhibitors have entered clinical trials. However, current side effects may limit the use of these compounds in people. In clinical trials, roxadustat showed a strong thrombosis signal even when compared to epoetin alfa [128], a drug for which thrombosis is a labeled adverse drug reaction. Therefore, a question remains as to whether there are still potential problems with the use of PHD inhibitors to stabilize HIF-2α, especially if such drugs need to be taken for long durations.

Given constitutive expression of HIF-1α and HIF-2α in RCCs with VHL mutation, HIFα inhibition has been of therapeutic interest. In a human trial treating clear cell renal cell carcinoma (ccRCC), HIF-2α was inhibited successfully by PT2385 albeit non-selectively in non-tumor tissue and ccRCC metastatic tumors [129]. Interestingly, prolonged PT2385 treatment resulted in resistance via a mutation in *EPAS1* which is hypothesized to interfere with drug binding [129]. The most common adverse event for HIF-2α inhibitors is likely anemia because erythropoietin (*EPO)*, which stimulates red blood cell production, is driven by HIF-2α expression [130,131].

Given that HIF-2α is associated with worse tumor outcomes in some models [62,76,83,96,97] and have better outcomes in others [9,59,71,94,95,101], future work should first focus on the therapeutic consequences of HIF-2α inhibitors and stabilizers in pre-clinical and translational models to best understand how targeting HIF-2α may lead to clinical benefit. Second, more research should focus if and how clinical manipulation of HIF-2α is safe, especially for chronic treatment.

We hypothesize that outcomes differ in these models for multifactorial reasons. Several studies that report worse outcomes associated with HIF-2α rely heavily on correlation than mechanism [76,96]. When the mechanism is unexplored, HIF-2α expression could also be interpreted as a protective mechanism rather than a negative indicator for prognosis. In addition, there is likely potential confounding variables in these studies, the most likely being hypoxia. For example, HIF-2α in TAMs correlated with increased breast tumor microvessel density [96] but so does whole tumor HIF-1α [PMID: 26079100] both of which are stabilized by hypoxia. Another possibility for discordance is compensatory immunosuppressive immune cell phenotypes. For example, Imtiyaz et al. reported alternative activation of macrophages increases canonical M2 marker, arginase 1 activity, independent of HIF-2α [62]. This suggests M2 phenotypes may persist despite HIF-2α inhibition due to immunosuppressive signaling stimulated by excessive cytokine production in tumors such as IL-4 or alternate hypoxia-induced transcriptional pathways independent of HIF-2α signaling such as Sp1 [132]. Importantly, the tumor model likely has influence. The murine tumors that were worsened by TAM HIF-2α were inflammation-induced HCC and colon tumors and a subcutaneous LLC tumor model. Given that TAM HIF-2α likely causes immunosuppression, the inflammation-induced HCC and colon tumors could be quickened because TAMs suppress cytotoxic immune cells that would be activated by this exogenous inflammation. As for the subcutaneous LLC tumor, it is likely that the tumor environment in the subcutaneous implant site varies drastically from the lung especially in tissue oxygenation. Interestingly, TAM HIF-2α expression is advantageous in all orthotopic murine tumor models reviewed here including breast cancer, skin cancer, and fibrosarcoma. The disadvantage of these models is the inherit dependence on the immortalized cancer line being implanted which likely varies significantly from the de novo formation of cancer which involves changes in tumor cells and stromal over tumor development in situ while orthotopic models rely heavily on stromal cell and immune cell recruitment after implantation. Understanding the differences among these preclinical models may shed light on targeting HIF-2α in patients.

## 8. Conclusions

HIF-2α expression is found in TAMs of several human tumors including bladder, brain, breast colon, liver, lung, ovarian, prostate, and PDAC tumors [110]. Yet, hypoxia-mediated TAM HIF-2α function remains largely unexplored. Despite few publications describing the hypoxia-dependent role of TAM HIF-2α, comprehensive review of HIF-2α in literature has provided insight into its role.

Preferential HIF-mediated transcription is likely dictated by the time and intensity of hypoxia experienced by cells within the tumor microenvironment. HIF-1α rapidly accumulates before HIF-2α during hypoxia, suggesting that HIF-1α may drive initial hypoxia responses [40] and macrophages accumulate higher levels of HIF-1α than HIF-2α when exposed to hypoxia, in vitro [109]. HIF-1α degrades faster than HIF-2α, suggesting a possible switch from HIF-1α to HIF-2α during hypoxic conditions [40,41,44]. This is further supported by data reporting HRE genes affected by acute hypoxia have promoter regions enriched with HIF-1α motifs while genes affected during prolonged hypoxia have more HIF-2α motifs [40,48,133–135]. However, these experiments neglect that tumor hypoxia is not static but rather cyclic [136]. Despite the literature reviewed here demonstrating that HIF-1α protein dominates in early hypoxia with a possible preferential switch from HIF-1α to HIF-2α during chronic episodes of hypoxia, it is not clear whether tumor hypoxia is adequately represented by “acute” or “chronic” conditions tested, in vitro [44]. Given HIF-1α expression rapidly degrades with re-oxygenation while HIF-2α remains elevated during re-oxygenation suggests HIF-2α may dominate in cyclic oxygen conditions such as those experienced in solid tumors [44].

However, Nanduri et al. reported HIF-1α is upregulated by intermittent hypoxia while HIF-2α is downregulated [80]. In fact, HIF-2α reduction is sequential with increased number of intermittent hypoxia cycles because of calpain protease activation [80]. Calpains may selectively target HIF-2α rather than HIF-1α because HIF-1α expression rapidly degrades with re-oxygenation, so calpain activation may deplete stabilized HIF-2α protein before de novo HIF-1α protein accumulation occurs resulting in dominating HIF-1α expression during intermittent hypoxia. Macrophages from mice exposed to 5 weeks of severe intermittent hypoxia have reduced *Epas1*/*Hif-2α* gene expression, also suggesting that chronic intermittent hypoxia may shift macrophage phenotypes away from Hif-2α-mediated expression [124]. No study to date has investigated intermittent hypoxia on HIF expression in macrophages, and we hypothesize this phenomenon persists in TAMs as our work and others continue to emphasize the negative consequences dictated by HIF-1α-mediated TAM phenotypes in pre-clinical models [9,137].

Primary human macrophages transfected with siRNA targeting HIF-2α expression also prevents hypoxic induction of *CTSB* and *SNX5* mRNA [60]. However, the functional relevance of these genes was unexplored. CTSB+ macrophages have been recently shown to repress anti-tumor immune response [138], suggesting that HIF-2α may drive this function. The sorting nexin (SNX) family’s role in TAMs, which includes SNX5, remains largely unexplored. However, SNX5 is essential for antigen processing and macropinocytosis, suggesting that HIF-2α may mediate TAM ability to process antigen in tumors [139]. More investigation of HIF-2α-driven gene expression is necessary to understand how these genes may be contributing to possible anti- versus protumor phenotypes in TAMs.

Several upstream signaling events regulate HIF-2α. Upstream signaling experimentation in RCC cells which have sustained HIF-2α protein expression revealed that estrogen receptor (ERβ) upregulates *HIF-2α* mRNA and protein through hypothesized transcriptional regulation at the *HIF-2α* promoter, suggesting partial dependence on the ER-pathway [68]. ERβ signaling in monocytes downregulates TNFα and IL-1β as well suggesting that ER-upregulation of *HIF-2α* may be associated with dampened inflammatory responses in macrophages [140].

During hypoxia, Reptin52 ATP-binding protein translocates from nucleus to cytoplasm and colocalizes with HIF-2α upon ERK1/2 inhibition in HeLa cells, suggesting that Reptin52 may reduce HIF-2α nuclear activity by a non-canonical PHD-VHL-proteasome independent mechanism [74]. This may be an attempt to dampen macrophage inflammation as transcriptome analysis in macrophages suggests that Reptin52 (RUVBL2) is an integral component of macrophage pro-inflammatory responses including NO production [141]. Myeloid HIF-2α suppresses NO production by competitive usage of L-arginine away from HIF-1α-driven iNOS-generated NO [62]. Considering that HIF-2α expression in macrophages dampens NO [56,62], Reptin52 may be functioning antagonistically against HIF-2α for NO production. Further supporting ERK1/2 regulation of HIF-2α, HIF-2α is directly phosphorylated by ERK1/2 in human hepatoma cells, and MEK inhibition upstream of ERK shifts hypoxia-stabilized nuclear-localized HIF-2α protein to the cytoplasm reducing HIF-2α-mediated gene expression [79].

## 9. Future Directions

There is a severe deficiency in macrophage HIF-2α-driven gene expression data available in the literature and further investigation is warranted. Tausendschon et al. reported *ADM* is regulated by HIF-2α and not HIF-1α in hypoxia-treated human macrophages [47]. *ADM* is important in TAMs for inducing angiogenesis and may explain why HIF-2α is associated with tumor microvessel density [96,111,142]. For example, TAM-derived ADM induces angiogenesis in murine melanoma [142]. ADM has also been linked to lymphangiogenesis which facilitates the dissemination of multiple cancer types; however, whether this is mediated by TAMs warrants further investigation [143–145]. ADM is also involved in cell proliferation and inflammation [146,147]. In addition to hypoxia driving *Adm* mRNA transcription in murine macrophages, a variety of inflammatory stimulants can also drive ADM production in immortalized murine macrophages including phorbol ester, retinoic acid, LPS, and IFN-γ [148]. In contrast, dexamethasone, hydrocortisone, estradiol, and TGF-β reduces ADM production in murine macrophages [148]. Whether HIF-2α is indispensable in these experiments remains unexplored.

Identification of HIF-2α-mediated Phd3 in RCC suggests a possible negative feedback mechanism as PHD3 preferentially targets HIF-2α and not HIF-1α for degradation [66,71]. This mechanism may persist in macrophages because *Phd3*/*Egln3* is also induced by hypoxia in mouse macrophages [47] and our laboratory found *Egln3* was the highest upregulated gene in murine BMDMs after hypoxia (0.5%, 24 h) in comparison to BMDMs at room oxygen. If this is a negative feedback mechanism, it may explain why HIF-1α dominates over HIF-2α in TAM phenotype in our orthotopic murine breast tumor model [9]. Macrophage HIF-1α deficiency decreased blood vessel density, improved vessel perfusion, increased tissue oxygenation, and permitted chemotherapy response compared to controls while macrophage HIF-2α deficiency exacerbated the control mouse phenotype. While this suggests a suppressive effect of HIF-2α on HIF-1α, it may also suggest that without HIF-1α present, the subtlety of HIF-2α-driven TAM phenotypes which are negatively regulated via feedback mechanism by PHD3 can now be observed.

RNA-sequencing of wild-type versus HIF-2α-deficient TAMs in murine breast tumors demonstrated 3 more genes *Spint1, IL-10*, and *Depdc7* were downregulated by HIF-2α knockout and had an identified HIF-2α (not HIF-1α) binding site using previously published ChIP-seq data sets [47]. Further investigation of Spint1, demonstrated HIF-2α-deficient BMDMs produce less Spint1 tumor suppressor than controls regardless of oxygen concentration resulting in faster orthotopic breast tumor growth, suggesting a paracrine role for HIF-2α in tumor suppression [59]. As for IL-10, microglia treated with IL-4, associated with an anti-inflammatory phenotype, suggested calcium signaling protein, Cav2.2 may block HIF-2α-driven expression of IL-10 [88]. Calcium availability may play an unexplained role of HIF-2α-driven expression of IL-10 in TAMs. Understanding this pathway is important as calcium signaling plays a major role in tumor immune cell function, cancer cell migration, and resistance to anti-tumor therapy [149–151]. The Dishevelled, EGL-10 and Pleckstrin (DEP) Domain-Containing Protein DEPDC7 has diverse roles in spatial and temporal signal transduction by recruiting proteins to the plasma membrane [152]. More recently, D’Andrea et al. reported DEPDC7 facilitates NF-κB activation in HEK-293 cells [153]. Liao et al. showed DEPDC7 inhibits cell migration and invasion hepatoma cells [154]. Perhaps, HIF-2α-induced *Depdc7* in TAMs limits migration and invasion in tumors and promotes NF-κB-mediated pro-inflammatory transcription to stimulate immune responses to control tumor progression. Given the suggested promiscuity of Depdc7, its role is likely involved in multiple signaling pathways unexplored in TAMs.

Understanding how the HIFs regulate adenosine receptors is important as adenosine signaling plays a major role in hypoxic conditions such as ischemia, inflammatory disease, or cancer [155]. Extracellular adenosine signals through four adenosine receptors (ADORs); ADORA1, ADORA2, ADORA2B, ADORA3 [155]. HIF-1α and HIF-2α have seemingly preferential modulation over the *Adora2b* and *Adora2a* genes, respectively. *Adora2b* is transcriptionally induced during hypoxia or inflammation by HIF-1α in endothelium and epithelial cells [156,157] while *ADORA2A* is a target of HIF-2α in human pulmonary endothelial cells [158]. In macrophages, *Adora2b* is seemingly regulated by HIF-1α, while both HIF-1α and HIF-2α regulate *Adora2a*. For example, Hadi et al. reported netrin-1, immuno-modulatory signaling molecule which signals through ADORAB2 peaks in aneurysmal human and murine macrophages and confers a pro-inflammatory expression signature [159], and Ramkhelawon et al. confirmed that HIF-1α induces netrin-1 in hypoxic macrophages, suggesting that HIF-1α indirectly promotes ADORA2B signaling by upregulating netrin-1 [160]. *ADORA2A* mRNA expression is downregulated by HIF-2α siRNA, not HIF-1α siRNA, in human MDMs, suggesting HIF-2α dependency. Given that deficiency of adenosine A2A receptors in myeloid cells (LysMcre/Adora2a*^flox/flox^*) reduces murine melanoma and increased tumor-suppressive MHCII and IL-12 expression in TAMs, HIF-2α may be driving an immunosuppressive, TAM phenotype that promotes tumor progression [161]. The anti-inflammatory functions of ADORA2A were first reported using genetic in vivo work by the Michail Sitkovsky laboratory which suggested ADORA2A functions in an endogenous feedback loop to dampen acute inflammatory responses [162]. Of note, studies by the Colgan [156] and Eltzschig [163,164] laboratories suggest hypoxia stabilized HIF-1A, not HIF-2, regulates ADORA2A, and these studies implicate a feedback loop that suppresses excessive inflammation and promotes ischemia tolerance and angiogenesis. For example, Eckle et al. found induction of ADORA2B during ventilator induced lung injury were diminished by alveolar epithelium *Hif1a* deficiency [165]. These data together suggest the macrophage anti-inflammatory phenotype that coincides with hypoxic induction of ADORA2B may be dependent on which HIFα subunit is dominating in that system.

Several works support HIF-associated macrophage polarization. Classical activation (M1-primed) of macrophages is linked to oxygen-independent preferential HIF-1α expression while alternatively-activated (M2-primed) macrophages have preferential expression of HIF-2α [55,89]. Importantly, macrophage polarization is accentuated by, but not dependent on, hypoxia. For example, Imtiyaz et al. reported alternative-activating cytokine IL-4 or hypoxia alone increase arginase 1 activity, a canonical M2 marker, independent of HIF-2α [62], suggesting that M2-priming of macrophages may increase HIF-2α, but HIF-2α does not confer the M2-like phenotype [62]. We wonder if the results would differ if the order was reversed, i.e., hypoxia priming then cytokine treatment, or if cytokines and hypoxia were given concurrently. Further investigation is warranted.

Importantly, review of various tumor models provides better understanding into TAM HIF-2α function. Several preclinical tumor models suggest a protective role for TAM HIF-2α with better outcomes in lung, fibrosarcoma, melanoma, breast tumors [9,59,71,94,95,97,101]. Our work demonstrated stabilization of TAM HIF-2α is synergistic with GM-CSF to produce sVEGFR-1 dampening excessive angiogenesis, limiting melanoma growth, and tumor cell extravasation [71,94]. TAM HIF-2α likely limits excessive angiogenesis in multiple models as HIF-2α inhibition or myeloid HIF-2α deficiency increase microvessel density and increase tumor growth in lung and breast tumors [9,97]. We found TAM HIF-2α likely suppresses excessive angiogenesis by limiting the effects of TAM HIF-1α-mediated increases in microvessel density and lower oxygen tension underscoring that vessel density does not equate with vessel perfusion [9]. In addition to its angiogenic role, Susen et al. identified TAM HIF-2α driven Spint1 tumor suppressor, suggesting a paracrine role for HIF-2α in tumor suppression [59]. In opposition, inflammation-induced tumor models suggest a detrimental role for TAM HIF-2α with increased tumor foci [62]. Interestingly, these tumor models and the Susen et al. breast tumor models see decreased in TAM populations at end point, suggesting that this effect may persist across tumor types regardless of tumorigenesis [62]. In vitro work suggests that TAM HIF-2α may drive recruitment capacity. HIF-2α-deficient BMDMs have reduced in vitro migration and invasion to chemoattractant CSF-1 [62]. CSF1 and pro-inflammatory cytokine IL-6 combination increase macrophage HIF-2α stabilization through PPARγ under normoxic conditions perhaps driving migration and invasion of macrophages [107]. Whether less TAMs are recruited to the tumor or TAMs are lost more rapidly in myeloid HIF-2α-deficient mice is yet to be determined, though reduction of TAMs spans several tumor types.

Of note, LysMcre/HIF-2α*^flox/flox^* mice used to investigate HIF-2α deficiency in TAMs is limited because HIF-2α deficiency is achieved in all lysozyme M expressing cells including monocytes/macrophages as well as neutrophils. This may be one reason differences are reported in various preclinical models. For example, pro-inflammatory pre-clinical models of DSS colitis and LPS-induced lung injury are seemingly dependent on HIF-2α in neutrophil populations preferably [86,87]. In immunosuppressive environments such as several solid tumors discussed here, the effects of HIFα deficiency reportedly dominate in macrophages. Nonetheless, all cell types experiencing HIFα deficiency should be evaluated to ensure conclusions are justified.

As we consider how TAM HIF-2α may be targeted clinically, we emphasize that cell-specific HIF knockout approaches limit the ability to elucidate HIF function in tumors where interactions among multiple cell types necessitate progression. For example, despite the advantageous effects of conditional HIF-2α KO in CAFs of PDAC, this effect did not translate during exogenous treatment. Treatment with HIF-2α inhibitor had the worse survival, even worse than the vehicle and IgG control [83]. This is likely due to the off-target effect of exogenous HIF-2α inhibition. Future work should highlight off-target effects to improve therapeutics aimed at HIF-2α.

## Figures and Tables

**Table 1.** HIF-2α regulated genes. HIF-2α regulated genes and the cell types in which the genes were reported.

HIF-2α Regulated Genes	Prior Literature
***ADM***	Induced by hypoxia in HUVECs [45,46] and **macrophages** [47]
*ANGPTL4*	Induced by hypoxia in HUVECs and human synoviocytes [45,46,49], regulated by HIF-2α in mouse embryonic fibroblasts [50]
*C1orf21*	Induced by hypoxia in HUVECs [45,46]
*MAGI1*	Induced by hypoxia in HUVECs [45,46]
*PTGIS*	Induced by hypoxia in HUVECs [32,33] and fibroblasts [51]
*LUCAT1*	Induced by hypoxia in *HUVECs [48]
*MIR210HG*	Induced by hypoxia in *HUVECs [48]
*BNIP3L*	Induced by hypoxia in *HUVECs [48]
***EGLN3***	Induced by hypoxia in *HUVECs [45,46,48] and mouse **macrophages** [47]
*SDF1*	Induced by hypoxia in human pulmonary ECs [52]
*CXCR4*	Induced by hypoxia in human pulmonary ECs [52]
*ICAM1*	Induced by hypoxia in human pulmonary ECs [52]
*TGFA*	Induced by hypoxia in human pulmonary ECs [52]
*WISP2*	Induced by hypoxia in breast cancer cell lines [53]
***Vegfa***	Induced by hypoxia in *retinal organoids [54] and * **BMDMs** [9]
***Arg-1***	Reduced in * **peritoneal macrophages** [55] from *HIF-2α^flox/flox^;Tekcre^+/−^* or **peritoneal macrophages** [56] treated with HIF-2α siRNA
*RAB11B-AS1*	Hypoxia-induced expression reduced in HIF-2α knockout human breast cancer cell lines [57]
*WNT5*	Luciferase reporter assays in AD-293 cells showed HIF-2α directly activates the *WNT5A* promoter [58]
***Spint1***	HIF-2α-deficient **BMDMs** produce less Spint1 than controls [59]
***ADORA2A***	mRNA expression downregulated by HIF-2α siRNA in **human MDMs** [60]
***CTSB***	Hypoxic induction prevented in **primary human macrophages** transfected with HIF-2α siRNA [60]
***SNX5***	Hypoxic induction prevented in **primary human macrophages** transfected with HIF-2α siRNA [60]
***IL-1β***	Hypoxic induction prevented in **human MDMs** transfected with HIF-2α siRNA [61]
***Cxcl2***	Reduced expression in hypoxia-treated murine **BMDMs** deficient in HIF-2α [62]
*Areg*	Reduced in murine ischemic cardiac tissue of *Hif2a^loxP/loxP^Myosin-Cre*+ [63]
*ERBB1*	Reduced in human cardiac myocytes using HIF-2α shRNA [64]

* Redundancy in HIF-1α/HIF-2α regulation. Genes regulated by HIF-2α in macrophages are bolded.

## Data Availability

No new data were created or analyzed in this study. Data sharing is not applicable.
